# Spatiotemporal dynamics reveal high turnover and contrasting assembly processes in fungal communities across contiguous habitats of tropical forests

**DOI:** 10.1186/s40793-025-00683-9

**Published:** 2025-02-15

**Authors:** Chieh-Ping Lin, Yu-Fei Lin, Yu-Ching Liu, Mei-Yeh Jade Lu, Huei-Mien Ke, Isheng Jason Tsai

**Affiliations:** 1 Biodiversity Research Center, Academia Sinica, Taipei, Taiwan; 2 Genome and Systems Biology Degree Program, Academia Sinica and National Taiwan University, Taipei, Taiwan; 3 Department of Microbiology, Soochow University, Taipei, Taiwan

## Abstract

**Background:**

The variation in fungal community composition within a single habitat space has been extensively studied in forest ecosystems. However, the spatial and temporal distribution of fungi across contiguous habitats, particularly at a local scale and in tropical regions, remains underexplored. In this study, we examined the fungal community composition across multiple habitats proximal to each other over two seasons in seven Fagaceae species in Taiwanese broadleaf forests. We tested how local spatial scale and habitat influence community assembly.

**Results:**

Using a metabarcoding approach, we sequenced ITS2 regions from 864 samples collected from four distinct habitats—leaves, twigs, litter, and soil. We identified 11,600 fungal amplicon sequence variants (ASVs), with community composition differing significantly between habitats proximal to each other. Generalized dissimilarity modeling (GDM) revealed that spatial distance, interacting with precipitation, was the strongest predictor of fungal turnover, particularly in the phyllosphere. Normalized Stochasticity Ratio (NST) analyses further highlighted contrasting assembly processes, with deterministic influences dominating in the phyllosphere habitat, while stochasticity prevailed in soil and litter. Random forest analysis accurately classified habitats based on ASVs’ relative abundances, with strong predictors were mostly habitat-specific ASVs prevalent in soil. Misclassified samples were due to secondary contact of fungi between adjacent habitats. Co-occurrence network analysis revealed more complex and deterministic networks in leaf and twig habitats, while soil was driven by stochastic processes and contained most habitat-specific ASVs. A *Cladosporium* sp. emerged as a keystone species, maintaining network stability across forests.

**Conclusion:**

This study reveals how local spatial variation and habitat shape distinct fungal communities in tropical forests, with deterministic processes dominating in some habitats and stochasticity playing a key role in others. We show extremely high turnover in fungal community are present over very short distances and that local fungal taxa are strong habitat predictors. These findings highlight the importance of studying coexisting habitats to gain a deeper understanding of fungal biogeography and ecosystem function.

**Supplementary Information:**

The online version contains supplementary material available at 10.1186/s40793-025-00683-9.

## Background

Forests are highly heterogeneous ecosystems, comprising a diverse array of distinct habitats. Even at the scale of individual trees, spatial heterogeneity is evident with various compartments ranging from fresh leaves to soil, each supporting distinct fungal communities. These fungi provide niche-specific processes as well as interface-mediated responses, which collectively contribute to the health and functioning of the forest ecosystem [1–6]. For example, phyllosphere fungi play essential roles in nutrient cycling and organic matter decomposition following the fall of leaves, transforming materials into absorbable forms for plants [7, 8]. While numerous studies have explored fungal diversity within specific habitats on a global scale [9, 10], investigating the spatiotemporal patterns of fungal communities across multiple proximal habitats at a local scale can reveal how these communities assemble, maintain, or partition themselves. Such an approach can yield crucial insights into the biogeography of microorganisms and ecosystem processes [11, 12].

Spatiotemporal variability of fungal communities has been extensively studied in other systems, particularly in soil environments where mycorrhizal fungi play key roles [11]. A nearly universal biogeographical pattern observed is the decay in community similarity with spatial distance [13, 14]. This distance-decay relationship can result from dispersal limitation, whereby the dispersal of species tends to decrease as the physical distance from the source increases [15]. In addition, geographically proximate locations often exhibit comparable environmental conditions, which serve to reinforce species similarity within these regions. Furthermore, the impact of these drivers on fungal communities varies across different habitats, leading to distinct patterns of community assembly [16]. These effects are inherently dependent on the spatial scale of the investigation [17]. At smaller spatial scale, stochastic processes may be dominant where environments are more homogenous. However, as spatial distance increases, environmental heterogeneity also increases (e.g., variations in vegetation, habitat chemistry, and climate), and community composition may then be more strongly determined with this heterogeneity [18]. Understanding whether community assembly is shaped by neutral processes or deterministic factors like niche differentiation is key to unraveling the spatiotemporal dynamics and ecological drivers of fungal communities.

Fungal community composition can vary significantly across different habitats of forest foreground, such as leaves, twigs, topsoil, and litter, even when these habitats are in close proximity. These habitats differ extensively in environmental factors such as chemical nutrient availability, but at the same time are connected via co-inhabiting fungi [19] and microbial-mediated ecotones [6]. Litter, for instance, can be particularly dynamic and experiences substantial seasonal changes even across very small distances [20] driven by the continuous influx of fresh litter [12]. The litter-soil interface is essential for nutrient cycling and organic matter decomposition, impacting soil fertility and plant health. Examining the fungal community of the forest ecosystem as a whole would enable us to quantify the diversity differences between different environments that were otherwise biased by methodologies [13] and to investigate the relationship between these environments and delineate ecosystem processes that affect single or multiple habitats simultaneously [19].

Tropical and subtropical forests, which cover 56% of the global forest area and support 42.8% of the world’s tree species, are among the most biodiverse ecosystems [19, 21]. Taiwan is a continental island with 60.7% of its land area covered by forests [22], which is more than double the global average of 30.2% and it ranks as the fifth highest nation in terms of tree density [23]. In particular, the broadleaf forests are home to a diverse range of Fagaceae plant and microbial species [24], yet studies on the mycobiome of Fagaceae species remain limited. Oak trees, which are keystone species in many ecosystems, play a crucial role in maintaining forest structure and function [25–27]. Despite the ecological importance of these trees, there has been a paucity of research on the fungal communities associated with the various habitats surrounding them, apart from mycorrhizal or soil fungi [28, 29]. Given Taiwan’s rich biodiversity, varied environmental conditions, and designation as a priority area for fungal conservation [30], it serves as an ideal place for studying fungal communities. Understanding the mycobiome of Fagaceae species in Taiwanese forests can provide valuable insights into the ecological processes governing these ecosystems and contribute to more effective forest management and conservation strategies.

In this study, we investigated fungal diversity across closely situated habitats—leaves, twigs, soil, and litter— associated with seven Fagaceae species in two broadleaf forests and a botanical garden in Taiwan to understand how these different environments influence the assembly and variability of fungal communities at a very local scale. Using a metabarcoding approach, we sequenced ITS3/ITS4 amplicons from 864 samples collected across four different habitats: leaves, twigs, litter, and topsoil from the same trees. By examining the spatiotemporal variation in mycobiomes at two time points, we sought to determine the influence of factors such as spatial distances, habitat type, season, and host species on fungal community composition. Our comprehensive analysis highlights the remarkably high turnover of fungal communities in the foreground of forests at small spatial scales, and underscoring the significant role of environmental factors in shaping these communities.

## Methods

### Sample collection

**Fig. 1:**
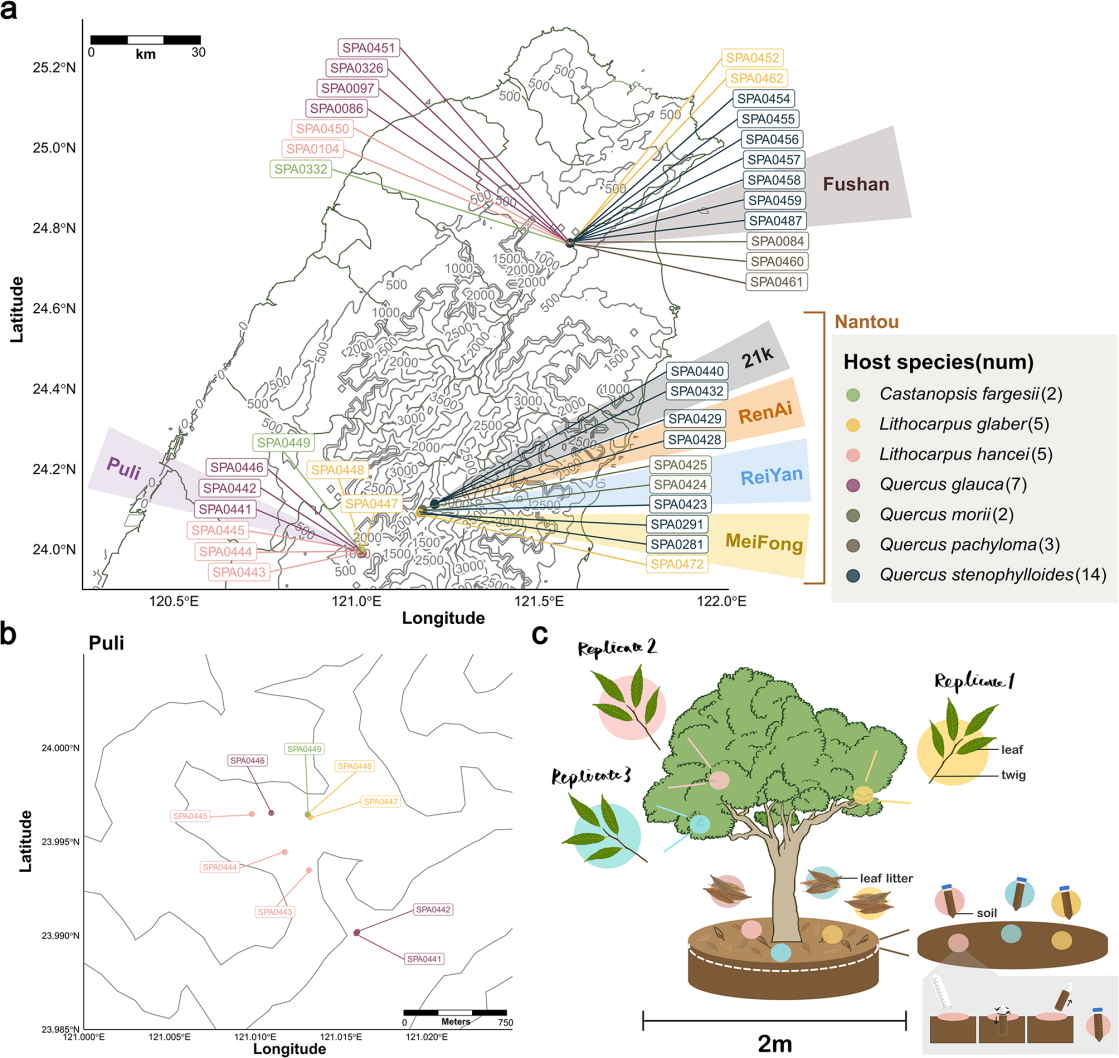
Sampled broadleaf forests in Taiwan surveyed for fungi associated with Fagaceae species. (**a**) Map of sampling sites. (**b**) Tree distributions in the Puli site. (**c**) A schematic diagram showing leaf, twig, leaf litter, and soil were sampled triplicates in each tree

To investigate the spatiotemporal variations in the forest mycobiomes, we collected samples from leaves, twigs, litter and topsoil of 38 trees belonging to seven Fagaceae species (*Quercus stenophylloides* *n* = 14, *Quercus glauca* *n* = 7, *Quercus morii* *n* = 2, *Quercus pachyloma* *n* = 3, *Castanopsis fargesii* *n* = 2, *Lithocarpus hancei* *n* = 5, *Lithocarpus glaber* *n* = 5) at two locations in Nantou County (Puli Township and Ren’ai Township) characterized by subtropical broadleaf forest dominated by Fagaceae and Lauraceae species [31], as well as the Fushan Botanical Garden in Yilan County, Taiwan (Fig. 1a and b). The Fushan Botanical Garden, established for the conservation and study of Taiwan’s subtropical forest ecosystems, features curated plantings of Fagaceae species and serves as a managed area with minimal human disturbance beyond its original design, preserving a semi-natural ecosystem. Unlike the Fushan Subtropical Forest Dynamics Plot (FDP), which is a natural forest research site, the botanical garden reflects a controlled environment [32]. Samples were collected at two time points per location, with Nantou samples harvested in April and October 2022, and Fushan samples in July and December 2022. The Fushan samples were designated as managed Fagaceae woodlands.

For each tree, triplicate samples of each habitat were obtained from different sides of the tree (Fig. 1c). The twigs, comprising approximately 20–30 leaves, were pruned with a sterilized tree lopper and placed into seal bags, with the leaves and twigs separated during laboratory preprocessing. Leaf litter (comprising 20–45 leaves) was sampled from the organic layer of soil, consisting mostly of late-stage decomposition material [33], while topsoil was sampled at a depth of 0–10 cm using 50 ml falcon tubes after the organic layer (“O” horizon) was removed. Originally 18 trees were chosen per survey site (a total of 36), but due to decay, two additional trees were substituted (SPA0446 and SPA0457 were replaced with SP0472 and SPA0487, respectively). A total of 864 samples were collected. All samples were refrigerated at 4 °C until extraction of genomic DNA (Supplementary Table S1). Abiotic variables associated with survey sites were collected from Climate Observation Data Inquire Service, Taiwan (Supplementary Table S2; https://codis.cwa.gov.tw/).

### Sample preprocessing and DNA extraction

The preprocessing steps for leaf, twig, and leaf litter samples followed the protocols detailed in our previous study [24]. The samples were cut into appropriately sized pieces to fit in the 500 ml bottles and suspended in 250 ml 1X PBS buffer (pH 7.4) containing 0.1% Tween 20. The suspension was subjected to sonication at 40 kHz for 20 min (DELTA ULTRASONIC CO. LTD, Make: DELTA, Model: DC400) and then shaken at 120 rpm for 1 h (FIRSTEK, Model: S-101) at room temperature. To remove large debris, the suspension was filtered through a 0.25 mm sterile mesh. Surface-associated genomic DNA was collected by passing the suspension through 0.22 μm PES membranes in a filtration cup (Jet Bio-Filtration Co., Cat. FPE214250). As a negative control of the preprocessing step, three samples with no substrate were processed for each batch to access the background contamination. This method primarily captures epiphytic fungi, targeting surface-associated microbes. Genomic DNA was extracted from the membranes using the DNeasy PowerWater kit (QIAGEN; Cat. 14900-50-NF) following the manufacturer’s instructions.

The topsoil samples were sieved using 2 mm steel mesh to remove plant debris, insects and rocks aiming to capture the entire fungal community within the soil. Genomic DNA was extracted from approximately 0.25 g of soil using the DNeasy PowerSoil Pro kit (QIAGEN, Cat. 47014) as instructed by the manufacturer. Homogenisation was performed with a Precellys 24 Touch homogeniser (Bertin Technologies, Cat. P002391-P24T0-A.0) at 5000 rpm for two cycles of 90 s, with a 15-second pause between cycles. The extracted DNA was quantified with Invitrogen Qubit 4 fluorometer (Invitrogen) and NanoDrop 1000 (ThermoFisher) and stored at -20 °C until library preparation.

### Amplicon library construction and sequencing

Amplicon libraries were constructed as previously described [34] using the forward primer ITS3ngs (5-CANCGATGAAGAACGYRG-3’) and reverse primer ITS4ngsUni (5’-CCTSCSCTTANTDATATGC-3’) [35, 36] to amplify the ITS2 region. The PCR mixture contained 50 ng of DNA extract, 2 µl of each 10 µM primer, 8 µl 5x HOT FIREPol Blend Master Mix (Solis Biodyne, Cat. 04-27-00115), 1 µl of 25 mM MgCl_2_ and ddH_2_0 to 40 µl. Extracted genomic DNA of *Saccharomyces kudriavzevii*, *S. paradoxus* and *S. cerevisiae* were used as single-species sample included as positive controls to assess the false positives in the sequencing and analysis. The thermal cycling conditions consisted of an initial denaturation at 95 °C for 12 min, followed by 35 cycles of denaturation at 95 °C for 20 s, annealing at 55 °C for 30 s, and extension at 72 °C for 1 min, finishing with a final cycle at 72 °C for 7 min. Biological replicates intended to be merged for sequencing were first pooled using equimolar dilutions of each. Amplicons were normalized to equal DNA quantity (approximately 25 ng) using SequalPrep™ Normalization Plate Kit (Invitrogen, ID: A1051001) according to the manufacturer’s instructions before pooling. The pooled library was concentrated to 10 ng/µl using AMPure XP (Beckman Coulter, ID: A63881). Each batch produced two plates of the library. Libraries were sequenced using the Illumina Miseq PE300 sequencing platform with equal molar pooling and 20% Phix spike-in. The sequencing was conducted by the NGS High Throughput Genomics Core of the Biodiversity Research Center, Academia Sinica, Taiwan.

### Statistical analyses

The raw sequencing data were imported and demultiplexed using *sabre* (v1.0; https://github.com/najoshi/sabre) allowing a 1 bp mismatch. Sequencing quality was examined using *FastQC* (v0.11.9; https://github.com/s-andrews/FastQC). Reads without primer sequences were detected and discarded with *usearch* (v11.0.667) [37]. Primer sequences were trimmed using *Cutadapt* (v4.4) [38]. The filtered and trimmed sequences were processed with the *Qiime2* (v2023.5.1) [39] pipeline to filter reads with a quality threshold of Qscore > 20 and to denoise into amplicon sequence variants (ASVs) based on algorithm of DADA2 [40]. Taxonomy for the ASVs was assigned using *constax* (v2.0.18) [41] with the UNITE Fungal database (v9.0) [42], and the trophic mode was annotated using *FUNGuild* (v1.2) [43].

Data processing and analysing as following were performed in the R-studio environment (v2024.4.1.748) [44]. Taxonomy levels were updated using package *rgbif* (v3.7.7) [45]. Background reads were subtracted based on the median read counts of ASVs observed in all negative controls. ASVs with relative abundances below 0.1% in each sample were filtered out. This cutoff was determined based on the relative abundance of contaminant ASVs identified in the positive controls, reflecting the threshold at which potential contaminants, including those arising from tag-switching or index-bleed, were observed. Preprocessed sequencing data (averaging 23,149 reads per sample) were rarefied to 5,000 reads for all analyses except co-occurrence network analysis, using phyloseq [46]. The preprocessing step excluded 57 samples. Figures were generated using *ggplot2* (v3.4.2) [47]. The sampling locations were annotated using *ggspatial* (v1.1.9; https://CRAN.R-project.org/package=ggspatial), *metR* (v0.14.0; https://github.com/eliocamp/metR) and *ggrepel* (v0.9.3; https://CRAN.R-project.org/package=ggrepel). Statistical significance test of the alpha diversity index was performed using *HSD.test* function in *agricolae* package (v1.3.7; https://github.com/myaseen208/agricolae). ASVs present in at least 25% of samples within a single habitat were classified as habitat-specific, while those occurring in 25% of samples across multiple habitats (e.g., 25% in both soil and leaf samples) were designated as ubiquitous ASVs. The *UpSetR* package was performed to visualise the habitat-specific and ubiquitous ASVs through the environments (v1.4.0; https://CRAN.R-project.org/package=UpSetR).

Sample heterogeneity was calculated with the vegdist function from the *vegan* package (v2.6-4; https://CRAN.R-project.org/package=vegan), focusing on samples with triplicates that underwent separate sequencing. The relative abundance of the ASV in the samples were transformed by square-rooting transformation. Comparisons were divided into three categories: ‘Within tree’ indicated the comparison between biological replicates of the same tree, ‘Within site’ referred to comparing samples from different trees in the same sampling sites and season and ‘Between sites’ showed the comparison of samples from different trees and sites in the same season (Spring of Puli vs. Spring of Nantou; Fall of Puli vs. Fall of Nantou). To examine the spatial and environmental predictors driving fungal community turnover, we employed Generalized Dissimilarity Modeling (GDM) using the *gdm* package (v1.6) [48] in R. Pairwise Bray-Curtis dissimilarities with square-root transformation of fungal communities in each habitat were used as the response variable, with geographic distance (derived from GPS coordinates), precipitation and temperature across multiple temporal scales (day of collection, three days, five days, seven days, 14 days, and 30 days) as predictor variables. Predictor variables were transformed into I-spline curves with function plotUncertainty with 1,000 times permutation, which models non-linear relationships and captures variations as turnover rates along environmental gradients [49]. P-values for each predictor were assessed using function gdm.varImp with 1,000 iterations of permutation, providing 95% confidence intervals for the predictors.

The normalized stochasticity ratio (NST) based on Jaccard distance was calculated to assess mycobiome assembly processes using the *NST* package (v3.1.10) [48]. A random forest model was used to classify samples into their respective habitats and to analyse ASV importance based on the mycobiome composition using package *randomForest* (v4.7.1.1; https://github.com/cran/randomForest/). Optimized parameters, including ntree, train/test ratio, mtry and minimum node size, were tuned using package *ranger* (v0.16.0; optimized parameters were as followed: mtry = 50; minimum node size = 4; ntree = 1,383) [49]. A total of 679 samples were transformed to relative abundance and subsequently divided into training set and testing set with an 80/20 ratio using function createDataPartition from package *caret* (v6.0.94) [50]. Cross-validation was used to prevent model overfitting, and model discrimination was visualized with the *pROC* (v1.18.5) [51] package.

### Network analysis

Triplicate samples from each habitat were initially pooled by rarefying to the minimum read depth among the triplicates and summing the rarefied reads. Leaf, twig, and litter samples were rarefied to 15,000 reads, while soil samples were rarefied to 10,000 reads. ASVs present in more than 50% of the habitat samples were retained by applying a 50% prevalence threshold to the rarefied data. Correlation indices were calculated using *FastSpar* (v1.0.0) [52, 53] with 100 iterations and filtered based on both significance (false positive adjusted p-value < 0.05) and strength (SparCC ≥ 0.6 or SparCC ≤ -0.6). Separate co-occurrence networks were constructed for each site, season and habitat type. The co-occurrence networks were visualized using *igraph* [54] with modularity and module identified using the *clustgreedyer_fast*_function [55]. To identify putative keystone taxa, we estimated the Zi (within-module degree z-score) and Pi (participation coefficient) values for each node using function within_module_deg_z_score and part_coeff of *brainGraph* (v3.0.0; https://CRAN.R-project.org/package=brainGraph) package, respectively. Nodes with Zi greater than 2.5 were defined as modular hubs; those with Pi more than 0.62 were identified as connectors; and nodes that satisfied both conditions of Zi ≥ 2.5 and Pi ≥ 0.62 were classified as network hubs.

## Results

### Fungal diversity across habitats and seasons in Taiwanese Fagaceae forests

We surveyed 38 trees from seven Fagaceae species across two natural Taiwanese broadleaf forests and Fushan botanical garden (Fig. 1). The survey was conducted at two time points and encompassed four habitats: leaf, twig, leaf litter, and topsoil, with triplicates for each tree resulting in a total of 864 samples (Fig. 1c). By amplifying and sequencing the ITS2 region, we quantified the relative abundances of mycobiomes across habitats and seasons. After quality filtering, denoising, merging and removing false positives, we obtained 17,222,990 sequences from an initial 46,317,215 paired reads and classified them into 11,600 amplicon sequencing variants (ASVs), averaging 69 ASVs and a median relative abundance of 0.40% per sample. The samples were further rarefied to 5,000 reads for subsequent analyses.

**Fig. 2:**
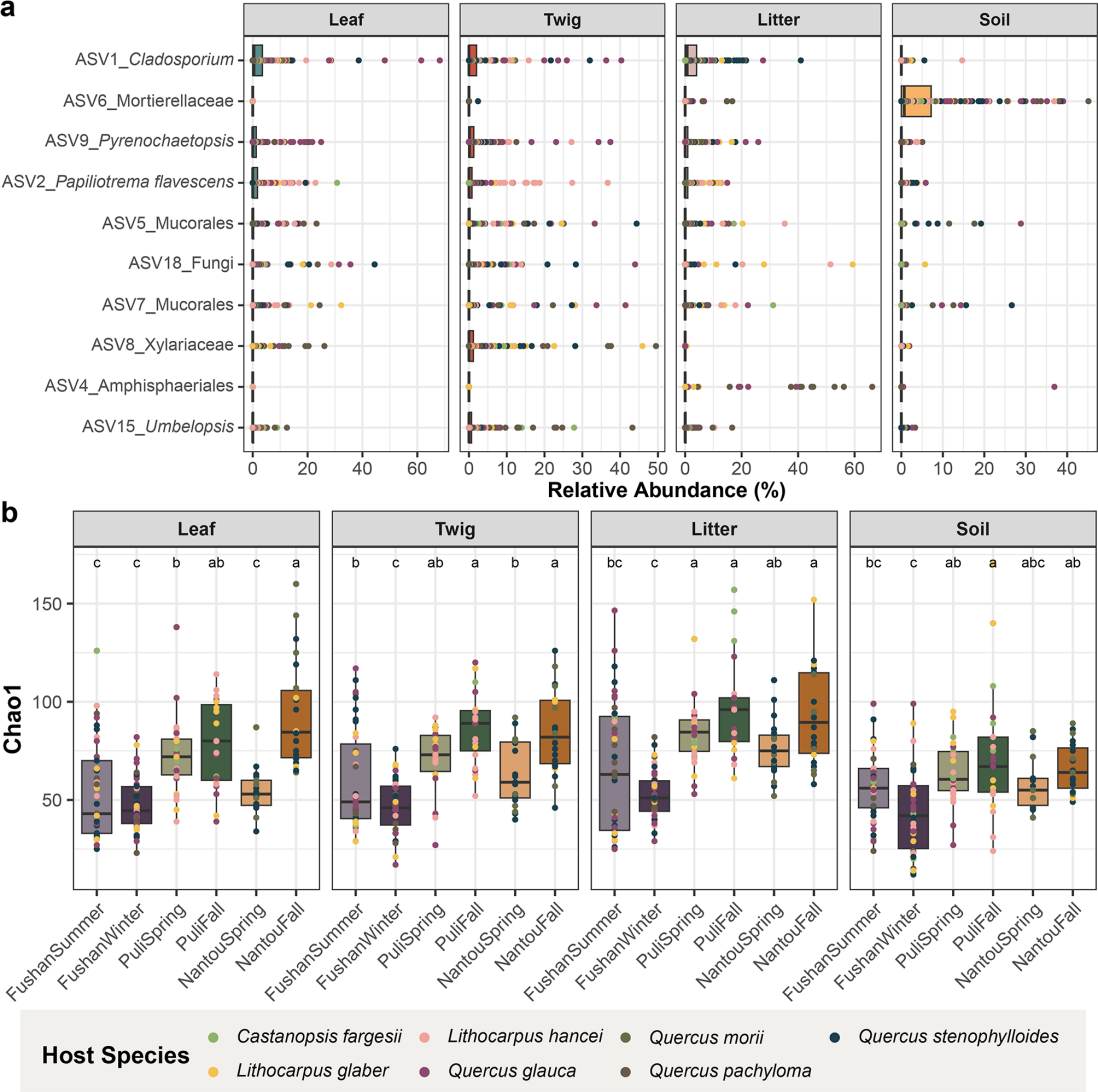
Fungal community diversity (**a**) The 10 most dominant ASVs’ relative abundance across samples. Each dot represents one sample, the color indicates the host species and the shape denotes the collection seasons; (**b**) Alpha diversity across habitats and seasons calculated using Chao1 index. The significant difference test was performed using Tukey’s HSD test, with different letters indicating statistically significant differences (*P* < 0.05). Dot color represents host species

The ASVs were classified into seven phyla with 5.8% remaining unclassified. The dominant fungal phylum was Ascomycota (56.3%), followed by Basidiomycota (26.9%), Zygomycota (10.6%), Glomeromycota (0.17%), Chytridiomycota (0.33%), and Blastocladiomycota (0.02%) (Supplementary Fig. S1). ASV1 was identified belonging to the *Cladosporium* genus and was the most dominant ASV present in 330 out of 679 samples with an average relative abundance of 5.48% (Fig. 2a). This ASV was particularly prevalent in leaf litter and leaves, with average relative abundance of 7.0% and 5.2%, respectively.

Alpha diversity, assessed using the Chao1 and Shannon diversity indices, revealed significant variation across habitats, with higher richness and evenness in litter (73 ± 27 ASVs; mean ± s.d.) compared to twig (65 ± 24 ASVs) and leaf (64 ± 26 ASVs) (Fig. 2b and Supplementary Fig. S2), while soil exhibited the lowest diversity (58 ± 22 ASVs). Seasonal variation also emerged as another significant driver of fungal communities (PERMANOVA; R^2^ = 0.09, *P* < 0.001). For instance, at the Fushan Botanical Garden, fungal richness and evenness declined in winter compared to summer, while in Nantou and Puli, Chao1 richness increased in fall compared to spring, though evenness remained stable (Fig. 2b and Supplementary Fig. S2a). There were no significant differences in both Chao1 and Shannon indices at the host species level, suggesting that host identity at a genus level may not play a major in determining fungal diversity in Fagaceae broadleaf forests. An exception occurred in Puli during the fall, where alpha diversity varied among host species, with *Castanopsis fargesii* exhibiting the highest diversity (Supplementary Fig. S2b). These findings suggest that while habitats and season are key determinants of fungal community diversity, host species may exert a more localized or context-dependent influence on alpha diversity. (Supplementary Fig. S2b).

### Spatiotemporal variation in fungal diversity and niche assembly of broadleaf forests

**Fig. 3:**
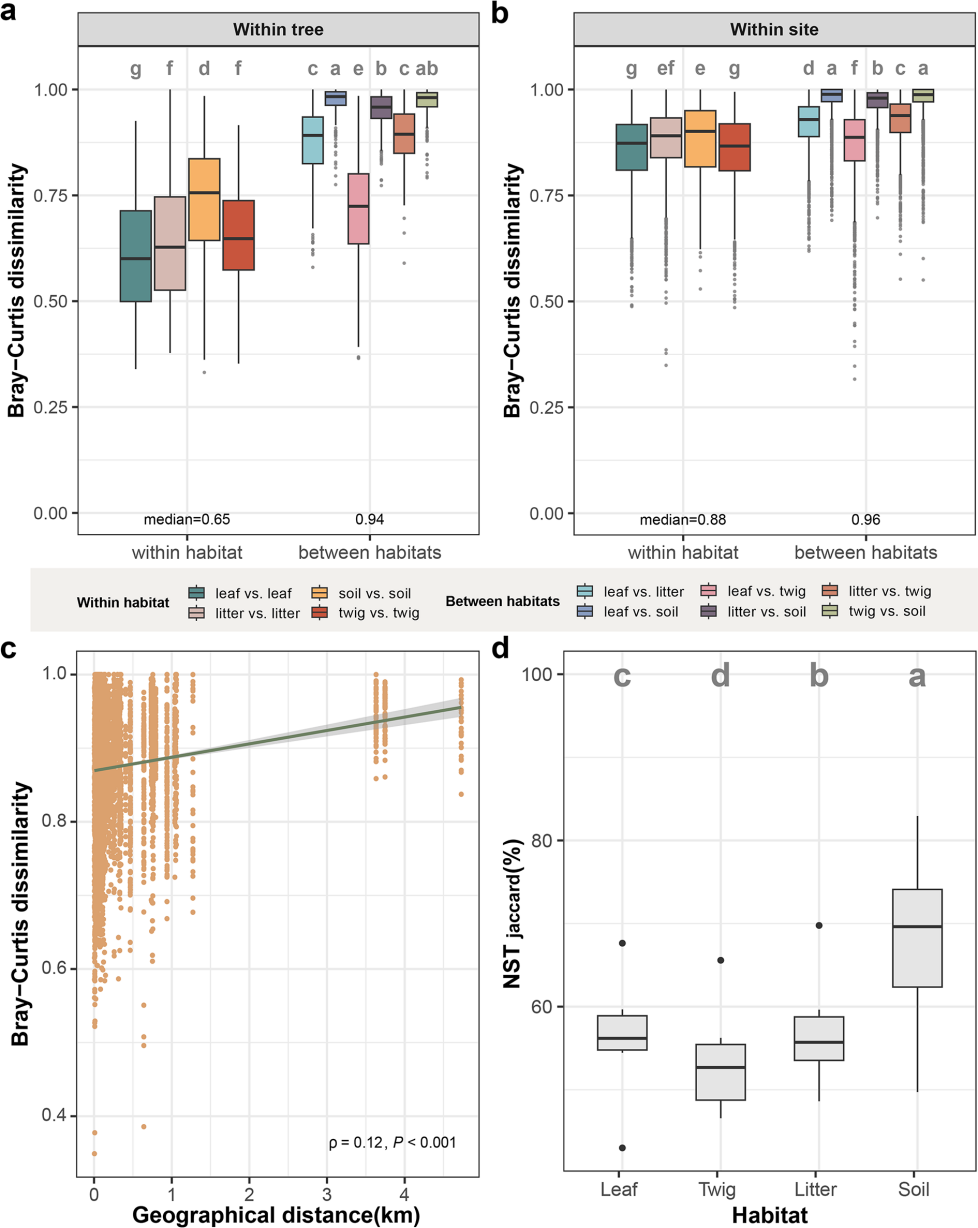
Dissimilarity and ecological stochasticity in the fungal community associated with Fagaceae species across habitats and geographical distance (km). Bray-Curtis dissimilarity of samples across habitats from the same tree (**a**) and different trees in one site (**b**). The colors indicate the habitat pairwise of two samples. (**c**) Distance-decay relationship of sample similarity of the same habitat between tree host species. Spearman’s rank correlation coefficient (ρ) and the corresponding significance are provided to demonstrate the strength and significance of the distance-decay relationship. (**d**) Community assembly processes are calculated by normalized stochasticity ratio (NST) based on Jaccard distance among habitats. The letters represent the statistically significant differences as estimated by Tukey’s HSD test (*P* < 0.05)

To investigate the spatial heterogeneity of fungal communities, we calculated the Bray-Curtis (BC) dissimilarity index for samples collected from the same trees, less than 2 m apart (Figs. 1c and 3a). Fungal community from the same habitat showed high dissimilarity even within individual trees (median BC = 0.65, Fig. 3a). Samples originating from the same habitats were more similar than those between different habitats (median BC = 0.65 vs. 0.94, *P* < 0.05, Tukey’s HSD; Fig. 3a). Site-specific factors influenced fungal turnover. For example, two *Quercus morii* and one *Q. stenophylloides* trees located in Rueiyan of the forest in Nantou exhibited the lowest BC values (Supplementary Fig. S3). Seasonal variation also contributed to higher BC dissimilarity, as evident in samples collected across different seasons (median BC = 0.85, Supplementary Figure S4). Among habitats, leaves exhibited the lowest dissimilarity (Fig. 3a and Supplementary Fig. S5). Overlap between litter vs. twig and litter vs. leaf, i.e., exhibiting low BC values, was also observed.

When considering distances between trees from the same forest site, samples from the same habitats remained highly dissimilar (median BC = 0.88; Fig. 3b) and a clear distance-decay relationship was observed (Fig. 3c). This relationship was stronger among trees of the same species compared to those of different species (Spearman’s ρ = 0.42 vs. 0.12, respectively; Fig. 3c and Supplementary Fig. S6), suggesting that host species played a significant role in determining fungal community composition at shorter distances, whereas communities between distantly located trees will be highly dissimilar, even if they belong to the same species. The distance-decay relationship was most pronounced in leaf vs. leaf comparisons, especially in Nantou during the Fall and Spring seasons (Spearman’s ρ = 0.43 and 0.53, respectively, Supplementary Fig. S7). In contrast, soil vs. soil comparisons displayed weaker or insignificant distance-decay relationships. As expected, samples from different habitats, trees and sites exhibited the greatest dissimilarity (median BC = 0.99; Supplementary Fig S8), suggesting that distinct habitat preferences strongly influence fungal community composition, limiting colonization across habitats despite physical proximity. In addition, we analyzed the normalized stochasticity ratio (NST) to evaluate the balance between stochastic and deterministic processes in fungal community assembly across different habitats and seasons (Fig. 3d). Twigs exhibited the lowest stochasticity (mean NST = 0.56), followed by leaves and litter, while soil had the highest stochasticity (mean NST = 0.68).

### Environmental predictors of forest mycobiome

**Fig. 4:**
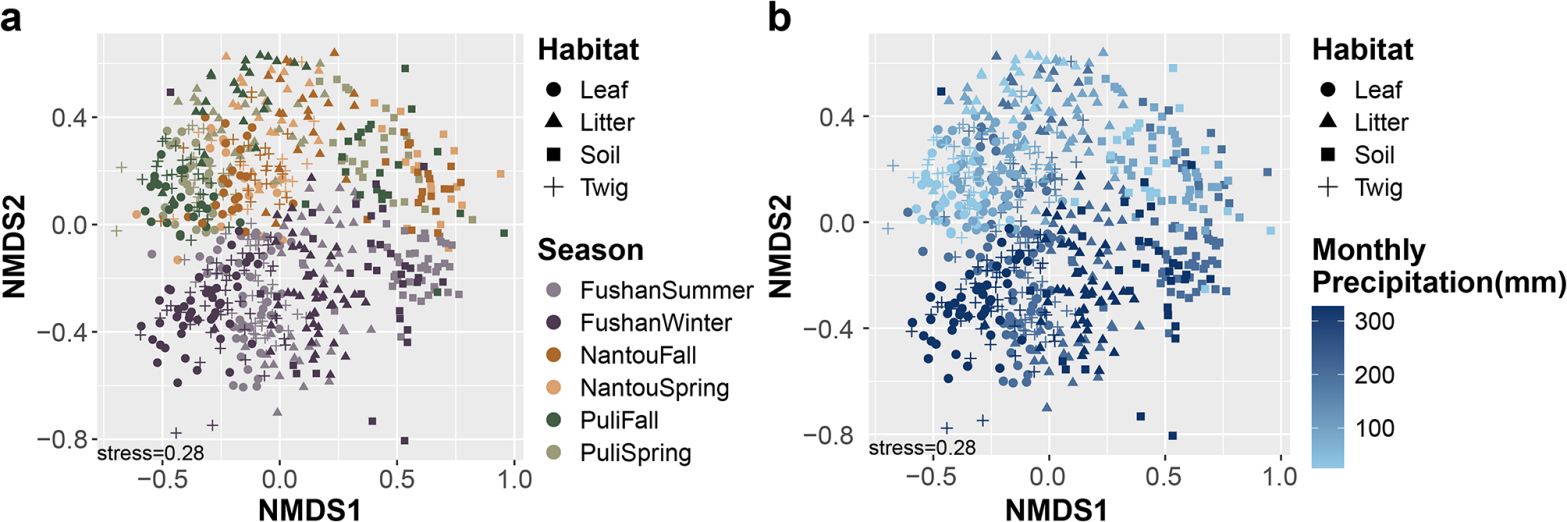
Fungal community differences among habitats, seasons and monthly precipitation. The plots were calculated using the Bray-Curtis distance, ordinated using Non-metric Multidimensional Scaling (NMDS) analysis. This analysis was performed at the genus level. (**a**) The shapes represent the habitat of the sample. The colors indicate the season of the samples; (**b**) The shapes represent the habitats and the colors represent the monthly precipitation (mm)

Fungal composition differences were analyzed using Bray-Curtis distance and visualized through Non-metric Multidimensional Scaling (NMDS) and Principal Coordinates Analysis (PCoA), with the first two principal components in PCoA explaining 14.3% of the variation in the dataset (Fig. 4 and Supplementary Fig. S9). Both NMDS and PCoA analyses revealed that samples collected from the same location but during different seasons significantly cluster together (PERMANOVA R² = 0.051, *P* < 0.001). Among habitats, NMDS analysis showed clear distinctions between soil and the other three habitats (leaf, litter, twig) (PERMANOVA R² = 0.037, *P* < 0.001) (Fig. 4a). Although host species had a significant influence on the fungal composition, the effect was relatively minor (PERMANOVA R² = 0.035, *P* < 0.001). Despite similar altitudes between the Fushan botanical garden (625–660 m) and the Puli forest (593–774 m), samples from these sites clustered separately.

Given the significant role of seasonality in shaping mycobiome composition, we explored the influence of climatic variables, focusing on relative humidity, temperature, and precipitation. Among these, monthly precipitation emerged as the most influential in determining mycobiome composition (PERMANOVA R² = 0.016, *P* < 0.001). PCoA based on Bray-Curtis distance, showed that PC1 (8.7%) effectively separated soil samples from the other habitats, while PC2 (5.6%) distinguished samples based on monthly precipitation levels, with higher precipitation associated with distinct fungal communities (Supplementary Fig. S9b). In contrast, daily precipitation had a weaker effect on fungal composition (PERMANOVA, R² = 0.007, *P* < 0.001), suggesting that prolonged rainfall, rather than short-term precipitation, plays a more critical role in gradually influencing fungal community composition.

**Fig. 5:**
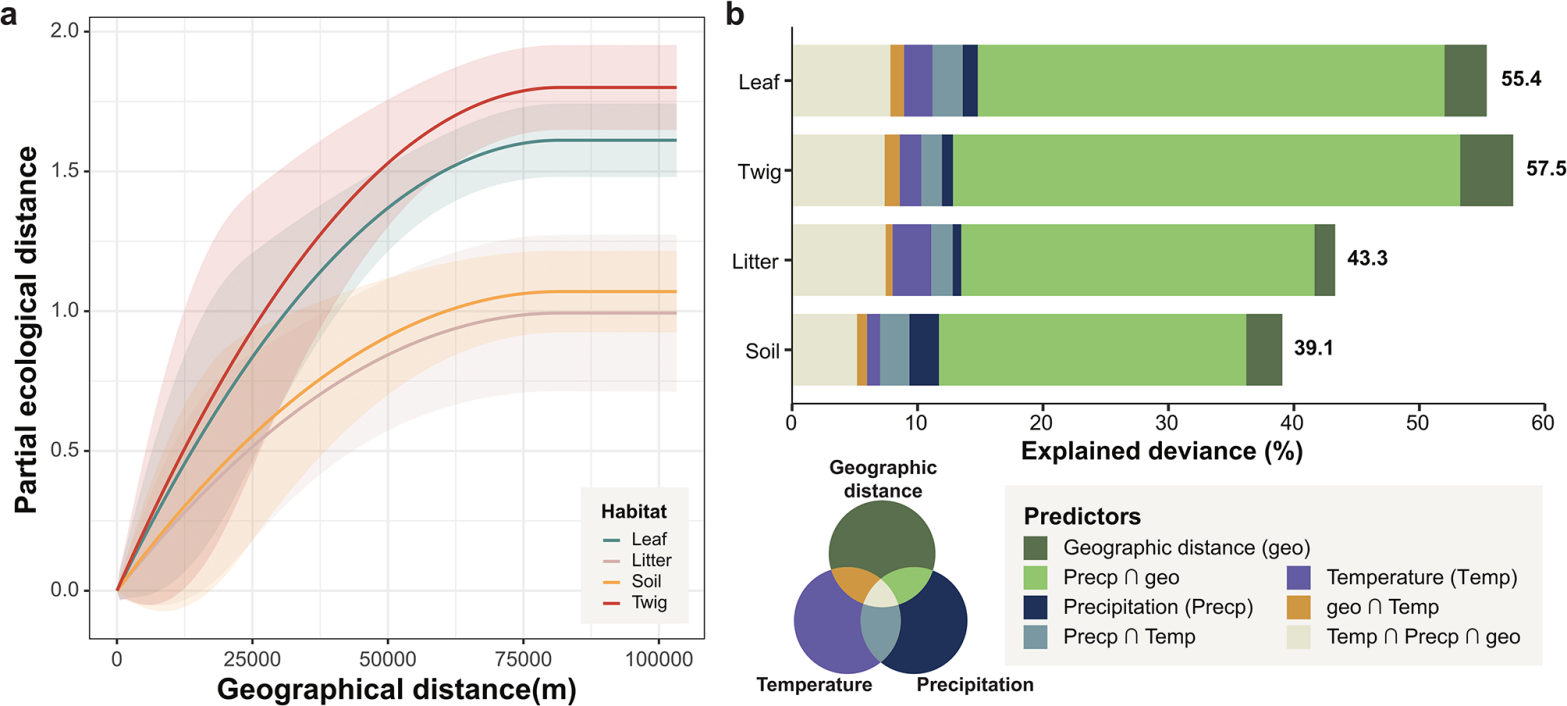
GDM analysis of spatial and environmental drivers of fungal community turnover across habitats. **a**) Fitted I-spline curves from GDMs illustrating the contribution of geographic distance on fungal dissimilarity across habitats. Shaded areas indicate standard deviation generated with 1,000 times permutation. Only significant predictors are displayed here with non-significant predictors shown in Supplementary Fig. S10; **b**) Partitioning of explained deviance (%) by predictors including temperature, precipitation, spatial distance and their interactions, with varying contributions across different habitats

We applied Generalized Dissimilarity Modeling (GDM) to quantify the spatial and environmental predictors driving fungal community turnover across habitats (Fig. 5), which explained 39.1–57.5% of the variation in dissimilarity (Supplementary Table S3a). Spatial distance emerged as the strongest determinant of fungal community dissimilarity when considered independently, with turnover occurring over small to intermediate spatial scales (Fig. 5a). Twig and leaf habitats exhibited the steeper I-spline gradients, indicating that fungal turnover in these habitats were more sensitive to geographic separation compared to soil and litter, which displayed a flatter response curve, consistent with their weaker distance-decay relationship. However, when environmental predictors such as temperature and precipitation were included, spatial distance became confounded with precipitation, with their combined effect explaining 38.0–55.8% of the variance in fungal dissimilarity across habitats (Fig. 5b). Although temperature and precipitation contributed comparatively little as standalone predictors (Supplementary Fig. S10), their influence was structured by site-specific factors, particularly in the phyllosphere, where fungal turnover exhibited greater sensitivity to environmental variation. Temperature alone explained a smaller proportion of the variance (1.1–3.1%).

### Distribution, trophic modes, and predictors of ASVs across forest habitats

**Fig. 6:**
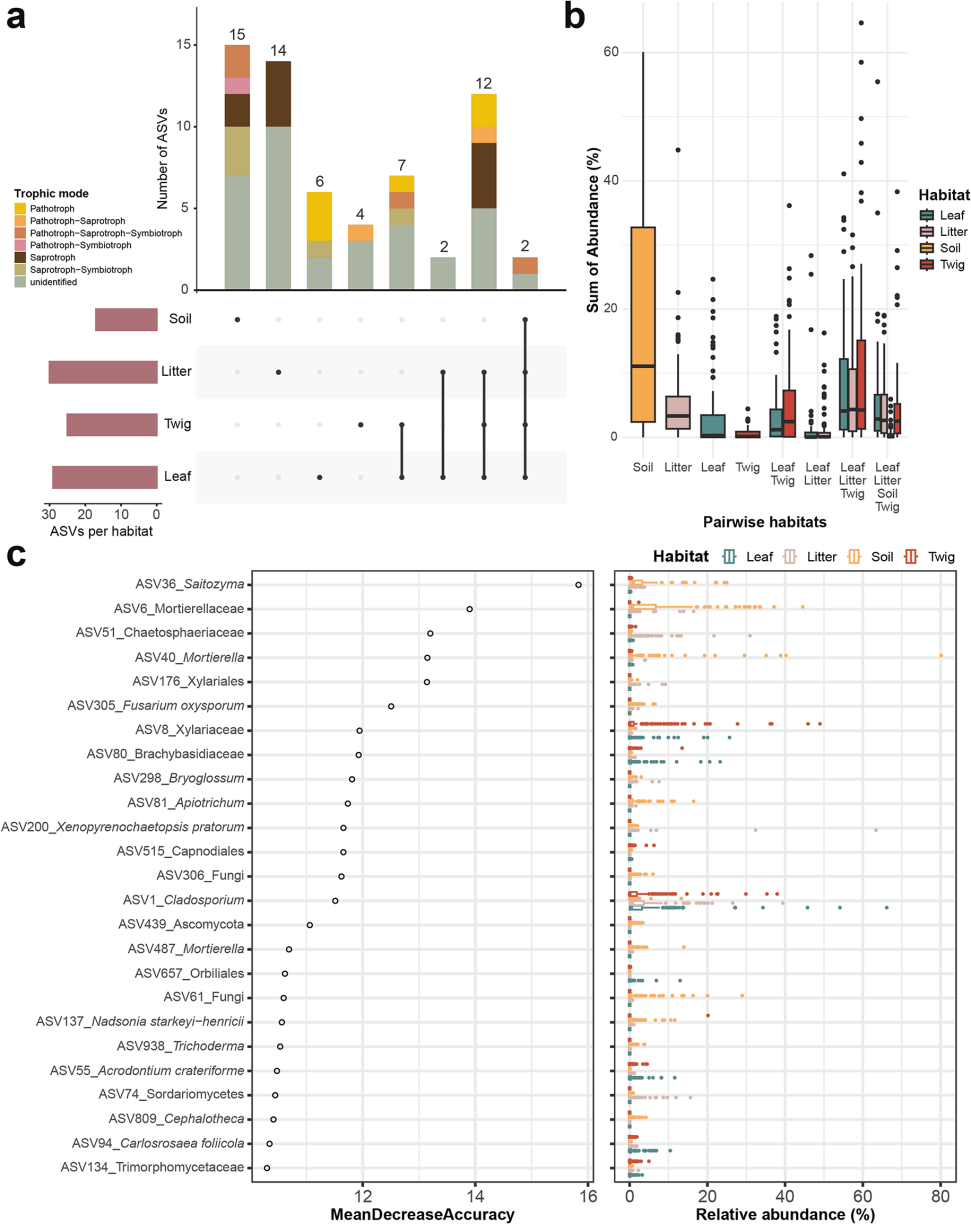
The endemicity of ASVs and their role in the random forest model. Number (**a**) and relative abundance (**b**) of habitat-specific and ubiquitous ASVs among habitats. Habitat combinations without sharing ASVs are not shown. (**a**) The bar color indicates the trophic mode of the ASVs; (**b**) Each boxplot represents the distribution of the total relative abundances of designated ASV across different habitats. For example, a data point in the soil category reflects the cumulative abundance of soil-specific ASVs within an individual soil sample. The box color represents the habitat. (**c**) Importance of ASVs in habitats classification and their relative abundance. The colors of boxes and dots represent its habitat.

To characterise the distributions of ASVs, we designated their status as habitat-specific and ubiquitous based on their presence across different habitats (see Methods; Fig. 6a and Supplementary Fig. S11) and annotated their trophic modes against the FUNGuild database [43]. Soil harbored the highest number of habitat-specific ASVs, with the saprotroph-symbiotroph trophic mode being the most prevalent, showing an average relative abundance of 3.4% across all soil samples (Fig. 6b). Although soil and litter are in close physical proximity, no ASVs were uniquely shared between these two habitats based on our filtering criteria. Only two ASVs-*Cladosporium* (ASV1) and *Pyrenochaetopsis* (ASV9)- were identified as ubiquitous across all habitats. In contrast, the above-ground habitats, encompassing leaves, twigs, and litter, shared 12 ASVs, which collectively account for approximately 8.89% of the relative abundance in each sample (Fig. 6b; Supplementary Fig. S11). Pathogenic fungi were predominantly found in above-ground habitats, with leaves exhibiting the highest abundances.

We employed random forest analysis to classify habitats based on ASV abundances and to assess the importance of specific ASVs in distinguishing between habitats (Fig. 6c). The model demonstrated strong predictive power, with an out-of-bag (OOB) error rate of 10.4% on training data and 10.3% on validation data (mean AUC = 0.98; Supplementary Fig. S12 and Supplementary Table S4a). Misclassification primarily occurred between leaves and twigs, with 30 out of 136 twigs misclassified as leaves (OOB error rate = 22.1%) and 15 out of 141 leaves misclassified as twigs (OOB error rate = 10.6%), consistent with the general low BC values in samples between these two habitats. Merging leaf and twig into a single category reduced the OOB error rate to 7.4%, achieving 100% classification accuracy for the combined category (leaf-twig) (Supplementary Table S4a). Interestingly, some litter and soil samples were misclassified as leaf or twig, but not vice versa. Specifically, 18 out of 142 litter samples were misclassified as leaf-twig, and 12 out of 126 soil samples were misclassified into other three habitats (Supplementary Table S4b). This pattern suggests the potential vertical movement of fungal communities from canopy to ground through falling leaves and twigs.

Several ASVs emerged as strong predictors of specific habitats. For example, ASV36 (*Salilomyza*) and ASV6 (assigned to the family Mortierellaceae) were key predictors for soil with relatively high relative abundances in soil samples (Fig. 6c), while ASVs such as ASV80 (assigned to the family Branchybasidiaceae), ASV8 (Xylariaceae) and ASV51 (Chaetosphaeriaceae) were more abundant in leaf, twig and litter samples, respectively, highlighting their role in shaping the unique fungal communities in the phyllosphere.

### Complexity and connectivity of co-occurrence networks across habitats

**Fig. 7:**
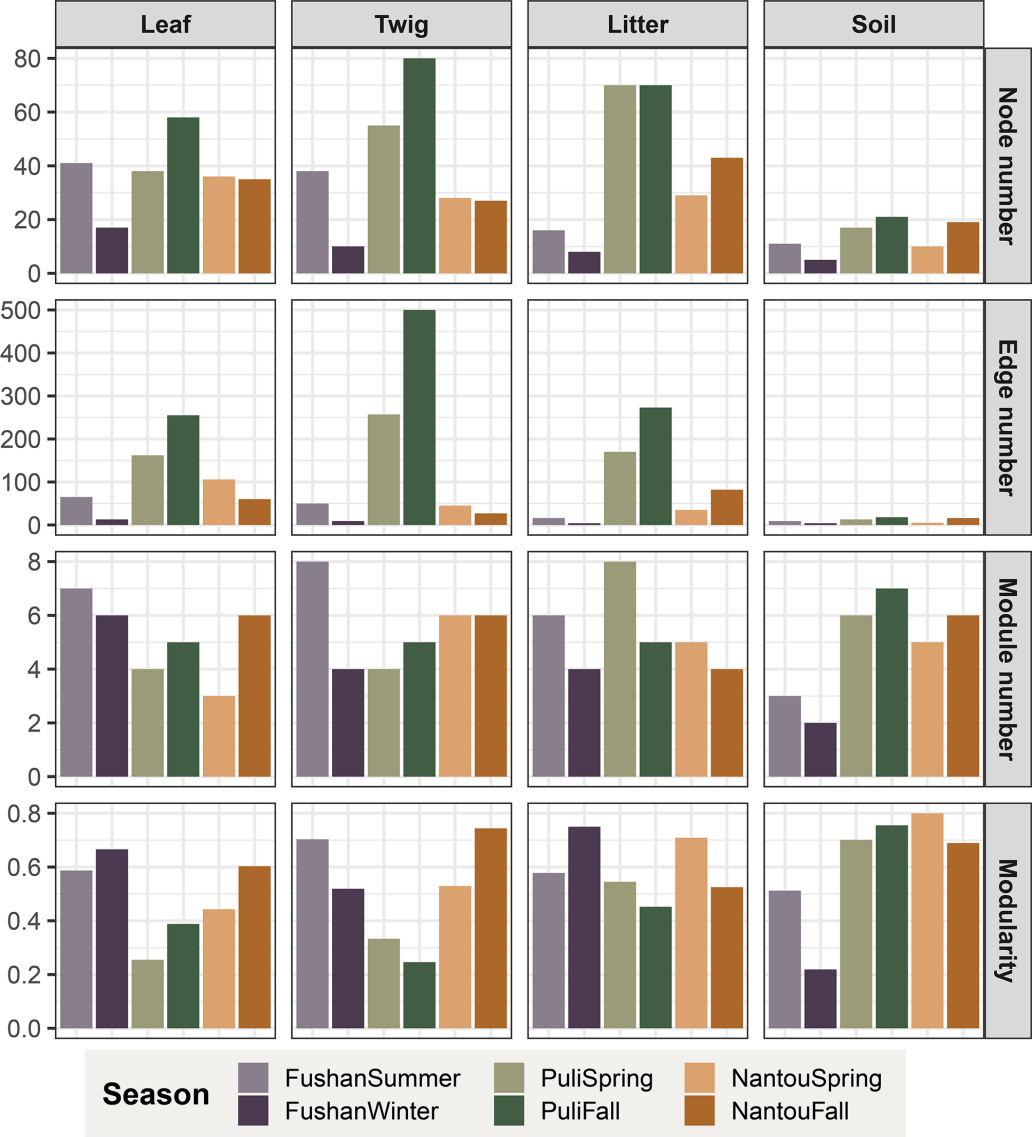
Complexity and connectivity of co-occurrence networks across seasons and habitats demonstrated by node number, edge number, module number and modularity

The co-occurrence network was constructed from 1,313,817 sequences representing 517 prevalent ASVs, revealing how different habitats and seasons influence fungal complexity and connectivity (Fig. 7; Supplementary Fig. S13). Significant variations in network size and complexity across different habitats and environmental conditions were observed. Soil networks were notably smaller and less complex than those of the other three habitats, with an average of 14 nodes (ASVs) compared to 39 in leaves, litters, and twigs. Seasonal comparisons revealed that network complexity decreased in winter compared to summer, with increases in the leaf of puli, twig of puli, litter, and soil, and decreases in the leaf and twig of Nantou from spring to fall (Supplementary Fig. S13), indicating that seasonal shifts, including changes in temperature and humidity, significantly impact fungal community co-occurrences. Modularity, reflecting how a network divides into distinct modules, varied significantly across habitats and seasons (Fig. 7 and Supplementary Fig. S13). For instance, in Fushan, the modularity of leaf and litter networks increased from summer to winter (modularity of summer vs. winter: leaf = 0.59 vs. 0.67, litter = 0.58 vs. 0.75), while twig and soil modularity decreased (modularity of summer vs. winter: twig = 0.70 vs. 0.52, soil = 0.51 vs. 0.22). Generally, the modularity and network complexity increased from spring to fall in leaves but decreased in litter, with twig and soil networks remaining relatively stable.

We found significant negative correlations between NST and both node and edge numbers (Spearman’s ρ= -0.41, *P* < 0.05 for nodes; Spearman’s ρ= -0.45, *P* < 0.05 for edges; Supplementary Fig. S14), indicating that as the number of nodes (ASVs) and edges (interactions) within a network increase, the influence of stochastic processes in community assembly decreases. Conversely, no significant correlations were observed between NST and the number of modules or modularity. The proportion of trophic mode nodes also varied across seasons, with an increase in the proportion of saprotroph nodes in soil and saprotroph and pathotroph-saprotroph nodes in litter during the fall (Supplementary Fig. S15), highlighting the role of environmental changes in shaping fungal community composition.

To assess the importance of individual nodes, we examined their connectivity within (modular hubs: Zi ≥ 2.5) and between (connectors: Pi ≥ 0.62) modules (see Methods; Supplementary Fig. S16), designating them as modular hubs or connectors based on criteria set in [56]. No shared modular hubs or connectors were found between seasons, even within the same location, and none were identified in soil or during winter. In leaf networks, eight connectors were identified, including two pathotroph-saprotroph-symbiotroph connectors (ASV9_*Pyrenochaetopsis*, Pi = 0.67, and ASV102_*Cryptococcus*, Pi = 0.65) and six unidentified connectors (Supplementary Table S5). The twig network had two connectors, while the litter network exhibited nine connectors (Supplementary Table S5) and one modular hub (pathotroph ASV614_*Dactylaria* acacia, Zi = 2.57). Only one putative keystone species—ASV1_*Cladosporium* (Pi = 0.66, Zi = 2.87)—was recognized as a network hub in the leaf of Puli during spring. Connectors were more frequently identified in leaves, twigs, and litter in Puli across both spring and fall, while in Nantou and Fushan, connectors were primarily observed in leaves or litter.

## Discussion

This study offers a detailed quantification of the spatiotemporal heterogeneity in fungal community composition within various above-ground habitats in tropical Fagaceae forests. By examining fungal communities in leaves, twigs, litter, and soil, we provide comprehensive insights into how different habitats influence fungal diversity at a both small and broad spatial scale. High BC dissimilarity (median 0.65) was observed among fungal communities from the same habitat, even when samples were collected from the same tree, consistent with previous studies that reported highly dissimilar fungal communities in litter fungi over short distances [20]. In addition, BC dissimilarity almost plateaued between fungal communities (median 0.94), suggesting a strong biogeography barrier resulting in disjunctive fungal communities. The GDM analyses further highlighted habitat-specific responses to environmental and spatial gradients, with leaf and twig community turnovers showing stronger associations with spatial distance, reflecting sensitivity to dispersal limitations, while litter and soil communities were less spatially constrained. Our analysis of the normalized stochasticity ratio (NST) further supports these observations, with twigs, litter and leaves showing lower NST values, indicating a stronger influence of deterministic processes, while soil exhibited higher NST values, reflecting greater stochasticity in these more dynamic habitats.

We uncovered distinct patterns in fungal community turnover across spatial scale and habitats. For example, in the Nantou region during spring, litter samples displayed a high correlation (Spearman’s ρ = 0.76), while no significant correlation was found in Puli during the same season. Soil fungal community turnover generally exhibited no or weak distance-decay relationships, which aligns with previous studies that found weaker distance decay in soil compared to other habitats [13, 57]. The exception observed in Fushan during summer may reflect site-specific factors driving community turnover at shorter distances. On a broader scale, environmental factors like precipitation exert a more significant role [11, 34]. Integrating these observations, the GDM analyses indicated that while dispersal limitation was the dominant factor influencing community turnover at small spatial scales, environmental filtering exerted a minor yet steady influence on fungal community assembly. The I-spline curves demonstrated a rapid plateau in turnover at small geographical distances (Fig. 5a), followed by a gradual rise attributed to abiotic factors along environmental gradients (Supplementary Fig. S10). Given the limited climate gradient focusing on comparing samples collected at small spatial scale in this study, the relatively minor role of environmental filtering in shaping fungal communities may be unsurprising. Nevertheless, this reinforces well with studies encompassing wider climatic and elevational ranges to fully capture the influence of abiotic factors on fungal community assembly [12, 58, 59]. In addition, the NST analysis further demonstrated that habitats with higher complexity and connectivity, such as leaf, litter and twig, were primarily influenced by deterministic processes, whereas more isolated or less complex habitats like soil were governed by stochasticity. These findings underscore the role of habitat-specific factors in shaping fungal community dynamics and highlight the importance of accounting for these factors across spatial scales when studying fungal biogeography in forest ecosystems. Contrary to the previous studies [60–62], host identity did not have a discernible impact on either alpha diversity or fungal composition. This discrepancy may be specific to Fagaceae or due to the limited phylogenetic diversity of seven sampled species, warranting the need for broader taxonomic sampling to better assess host effects on fungal communities.

Documenting fungal communities across multiple habitats at small spatial scales allowed us to identify instances of overlap, with approximately 6% of samples from one habitat being more similar to samples from another habitat. This overlap suggests potential pathways for fungal movement or shared environmental conditions that facilitate community convergence and reinforce the importance of spatial orientation and environmental context in the dispersal and establishment of fungal communities. For example, certain litter samples showed similarities with soil communities, likely due to the physical proximity and interaction between these habitats [20]. This pattern emphasizes the importance of microbial-mediated interfaces, such as the litter-soil interface, in shaping community composition [6]. Moreover, the presence of similar communities across distinct habitats highlights the potential role of dispersal processes and environmental filtering in shaping fungal communities [11]. Interestingly, such interactions can lead to misclassification of sample origin in the random forest analysis (error rate 10.4%), and we observed no misclassification in leaf and twig samples as litter or soil, possibly due to the reduced likelihood of upward transmission compared to horizontal movement across other habitats. In addition, the presence of airborne spores on surfaces may further influence fungal dispersal patterns and classification accuracy.

An unidentified *Cladosporium* (ASV1) serves as the sole network hub in the co-occurrence network analysis and a key habitat predictor in the random forest model (mean decrease accuracy = 11.5). ASV1 also emerged as the most dominant species in the study, particularly in leaf and litter samples, as indicated by studies that have revealed *Cladosporium cladosporioides* as a common species in the phyllosphere and on fallen leaves [63]. A subsequent investigation of *Cladosporium* strains isolated from lake environments further confirmed their ability to degrade various forms of humus, including laccase, lignin and triarylmethane [64]. The widespread presence of *Cladosporium* in these habitats highlights its potential role in leaf composition, nutrient cycling and organic matter decomposition, thereby emphasizing its ecological importance in phyllosphere fungal communities. In addition, *Saitozyma* (ASV36) and *Apiotrichum* (ASV81) were important predictors for habitat classification and were predominantly found in soil. This dominance is supported by prior findings from European Fagaceae forests [65], underscoring broader ecological importance beyond local habitats.

## Conclusion

By examining spatiotemporal heterogeneity across coexisting habitats, we reveal contrasting processes driving fungal community assembly in tropical broadleaf forests. Generalized Dissimilarity Modeling (GDM) underscored the predominant role of spatial distance, along with its interaction with environmental gradients—particularly precipitation—in shaping community turnover, with varying effects across habitats. Deterministic influences dominate in the interconnected leaf, twig and litter habitats, while stochasticity is more prominent in soil, highlighting the role of habitat features in shaping communities. The identification of *Cladosporium* sp. as a keystone species further highlights the ecological importance of certain taxa in maintaining network stability and community cohesion. Random forest analysis identified distinct fungal signatures, with misclassification linked to natural overlap between habitats. Our findings emphasize the importance of studying multiple habitats together for understanding fungal biogeography and underscore the need to explore habitat-specific drivers for ecosystem functioning and conservation in response to climate change.

### Data Availability

The demultiplexed Illumina data were deposited to NCBI under BioProject PRJNA1156365. The accession number of each sample were complemented in Supplementary Table S1.

## Electronic supplementary material

Below is the link to the electronic supplementary material.

Supplementary Material 1

Supplementary Material 2

## Data Availability

The demultiplexed Illumina data were deposited to NCBI under BioProject PRJNA1156365. The accession number of each sample were complemented in Supplementary Table S1.
