# Supplementary material for: Spatiotemporal dynamics reveal high turnover and contrasting assembly processes in fungal communities across contiguous habitats of tropical forests
Source: Environ Microbiome. 2025 Feb 15;20:23. doi: 10.1186/s40793-025-00683-9 (PMC11830174; doi:10.1186/s40793-025-00683-9)

Spatiotemporal dynamics reveal high turnover and contrasting assembly processes in fungal communities across contiguous habitats of tropical forests

Chieh-Ping Lin[1,2], Yu-Fei Lin[1], Yu-Ching Liu[1], Mei-Yeh Jade Lu[1], Huei-Mien Ke[3] and Isheng Jason Tsai [1,2]

[1]Biodiversity Research Center, Academia Sinica, Taipei, Taiwan
[2]Genome and Systems Biology Degree Program, Academia Sinica and National Taiwan University, Taipei, Taiwan
[3]Department of Microbiology, Soochow University, Taipei, Taiwan

Correspondence: ijtsai@gate.sinica.edu.tw

Supplementary Figures

Fig. S1 Relative abundance of fungal phyla across different habitats and seasons. Bar plots represent the fungal community composition at the phylum level, with colors indicating the specific fungal phyla. Samples were collected from four habitats (leaf, litter, soil, and twig) during different seasons (FushanSummer, FushanWinter, PuliSpring, PuliFall, NantouSpring, and NantouFall). Each bar corresponds to a different habitat, revealing seasonal and habitat-specific variations in fungal phyla distribution.

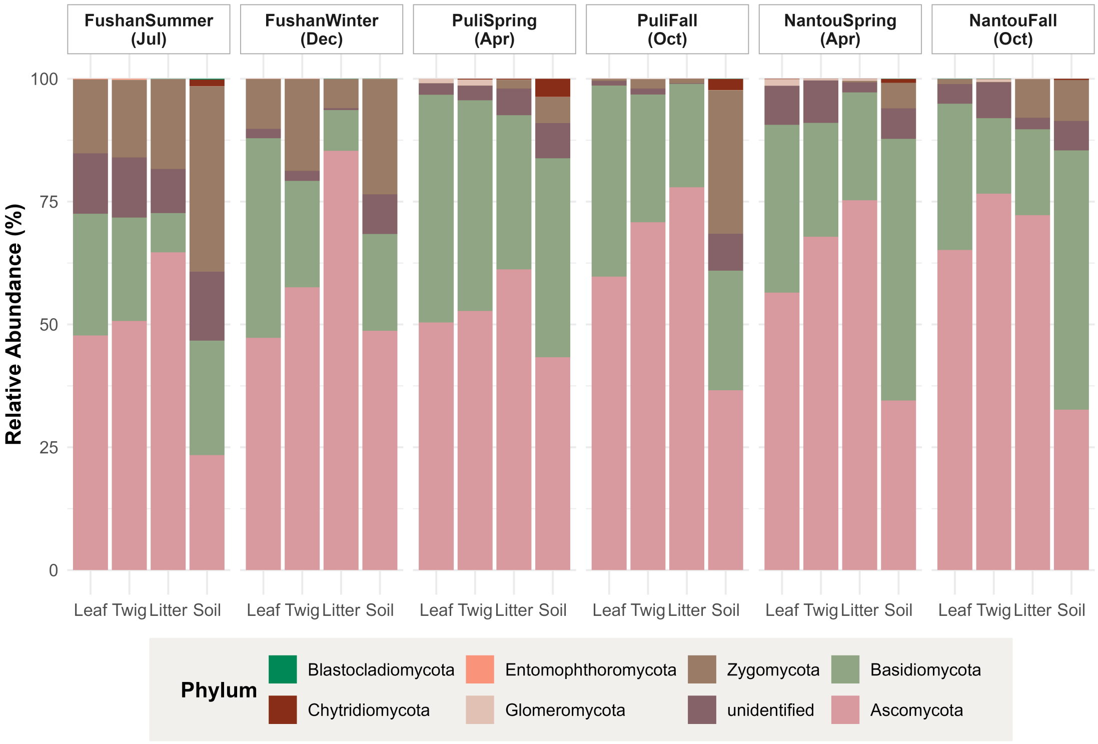

Fig. S2 Box plots of mycobiome alpha diversity across (a) habitats and (b) host species calculated using the Chao1 index and Shannon's diversity index. Dot colors represent different host species. Tukey's HSD test was performed to assess significant differences in diversity indices between groups, with different letters indicating statistically significant differences (P < 0.05).

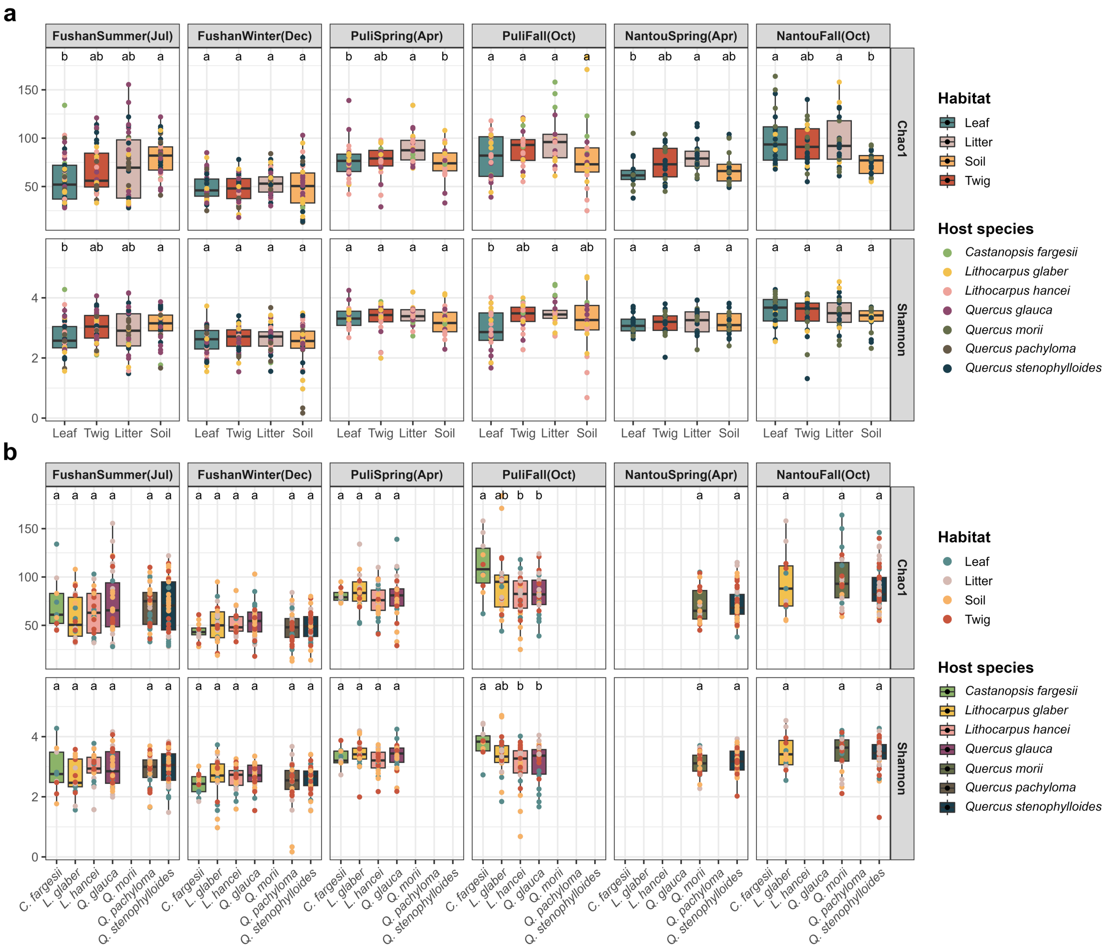

Fig. S3 Bray-Curtis dissimilarity between samples from the same tree and habitat across regions. Each point represents the dissimilarity index between sample pairs originating from the same tree. The colors indicate the host tree species for each sample. Different letters above the box plots indicate statistically significant differences based on Tukey's HSD test for all pairwise comparisons (P < 0.05).

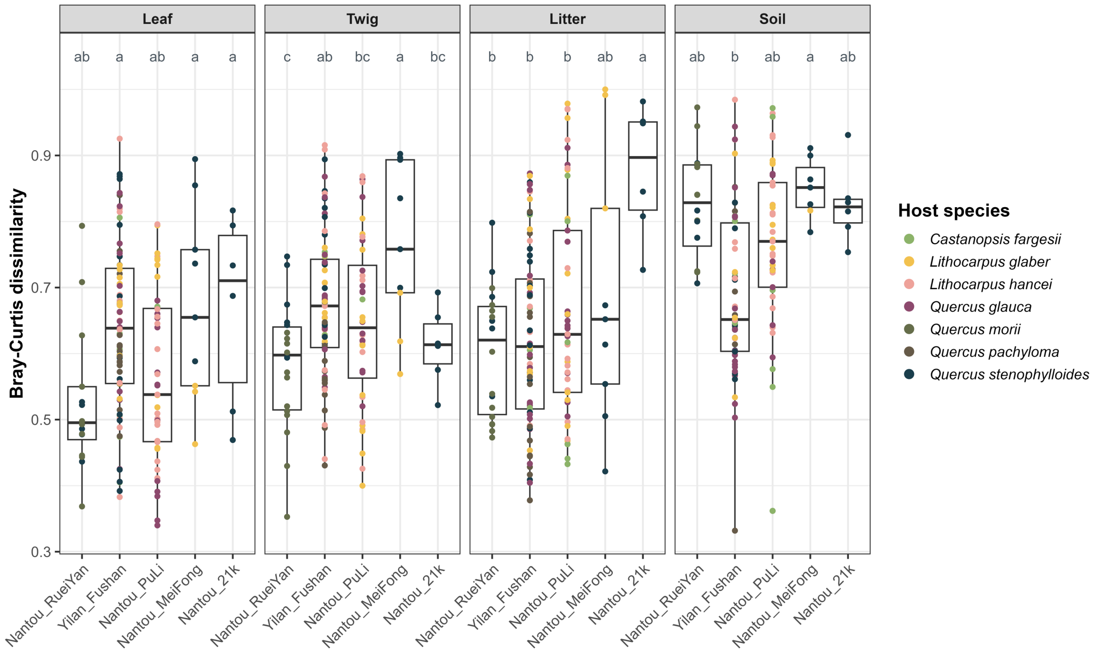

Fig. S4 Bray-Curtis dissimilarity between merged samples due to seasonality. The comparison of sites Nantou and Puli focused on spring against fall, whereas Fushan compared summer versus winter. The colors indicate the habitat for each sample. The letters above box plots represent statistically significant differences based on Tukey's HSD test ($P < 0.05$).

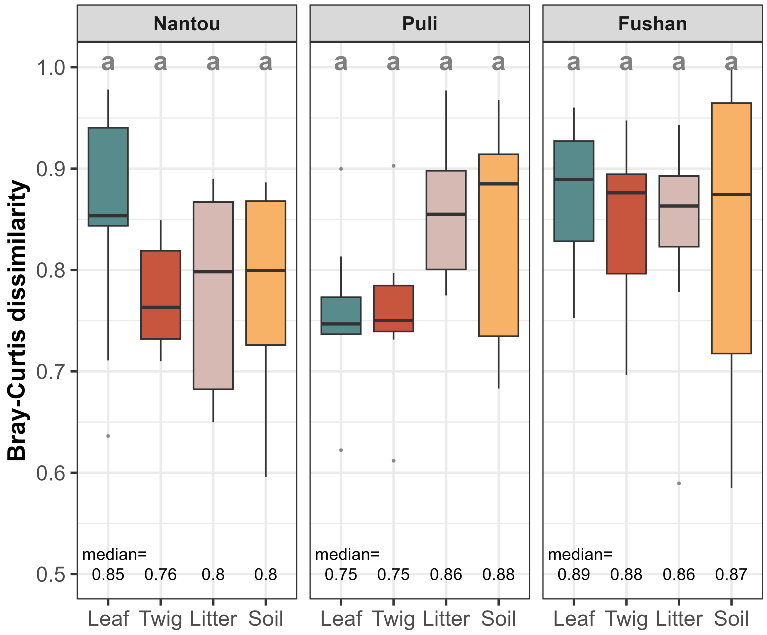

Fig. S5 Bray-Curtis dissimilarity between samples from the same habitat across forests and seasons. Each point represents the dissimilarity index between pairwise sample comparisons. The color of the point denotes the host species.

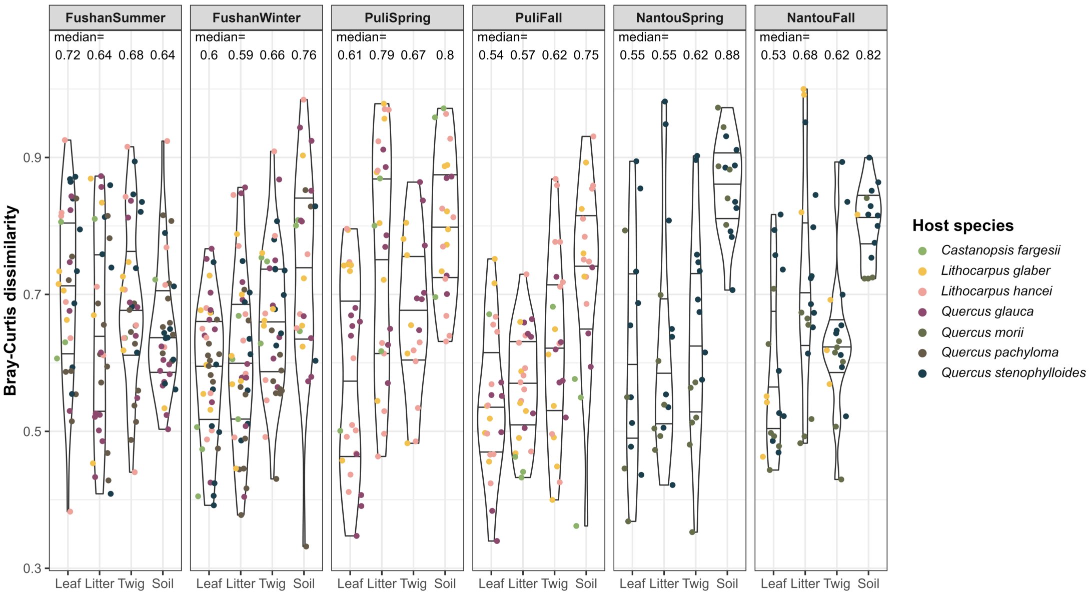

Fig. S6 Distance-decay relationship of sample dissimilarity between and within tree host species. The Bray-Curtis dissimilarity index is plotted against geographical distance (km) between sample pairs. The left panel shows the comparisons between different host species, while the right panel shows intraspecific host comparisons. Spearman's rank correlation coefficients (ρ) and associated significance values (P) are provided for both comparisons.

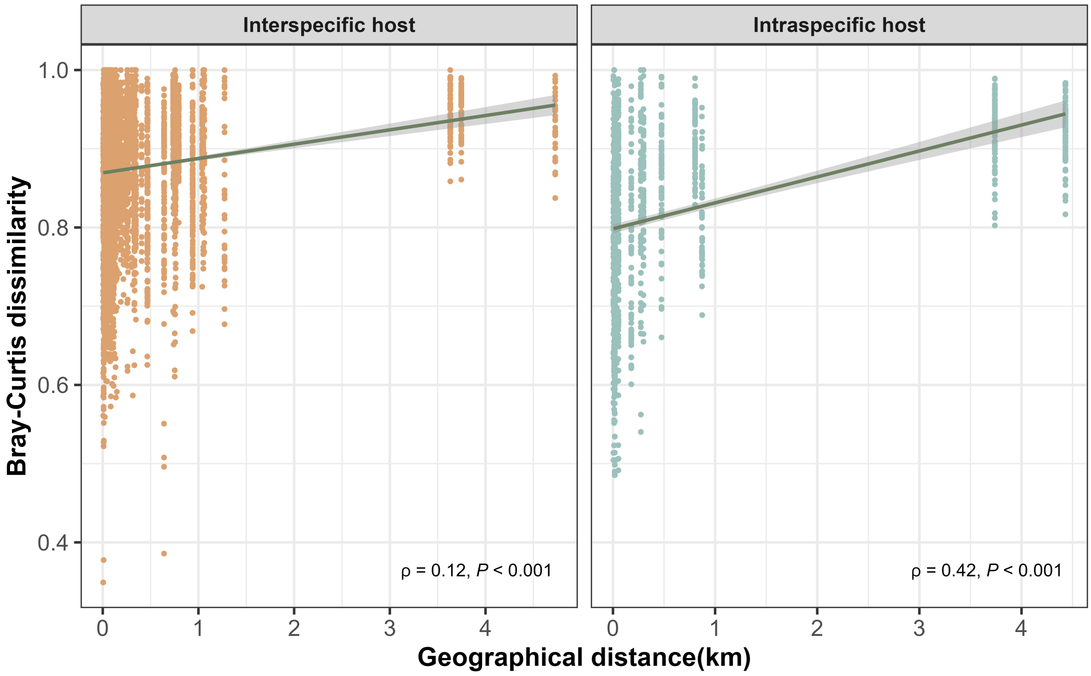

Fig. S7 Distance-decay relationship of sample similarity within site and habitat across seasons. The Bray-Curtis dissimilarity index is plotted against geographical distance (meters) between pairs of samples originating from the same habitats (leaf, litter, soil, and twig) across seasons. Each panel shows the distance-decay relationship for a specific season and habitat. Dot colors represent whether the host species of the two samples were the same (green) or different (orange). Spearman's rank correlation coefficients (ρ) and associated significance values (P) are provided for each comparison to indicate the strength and significance of the distance-decay relationship.

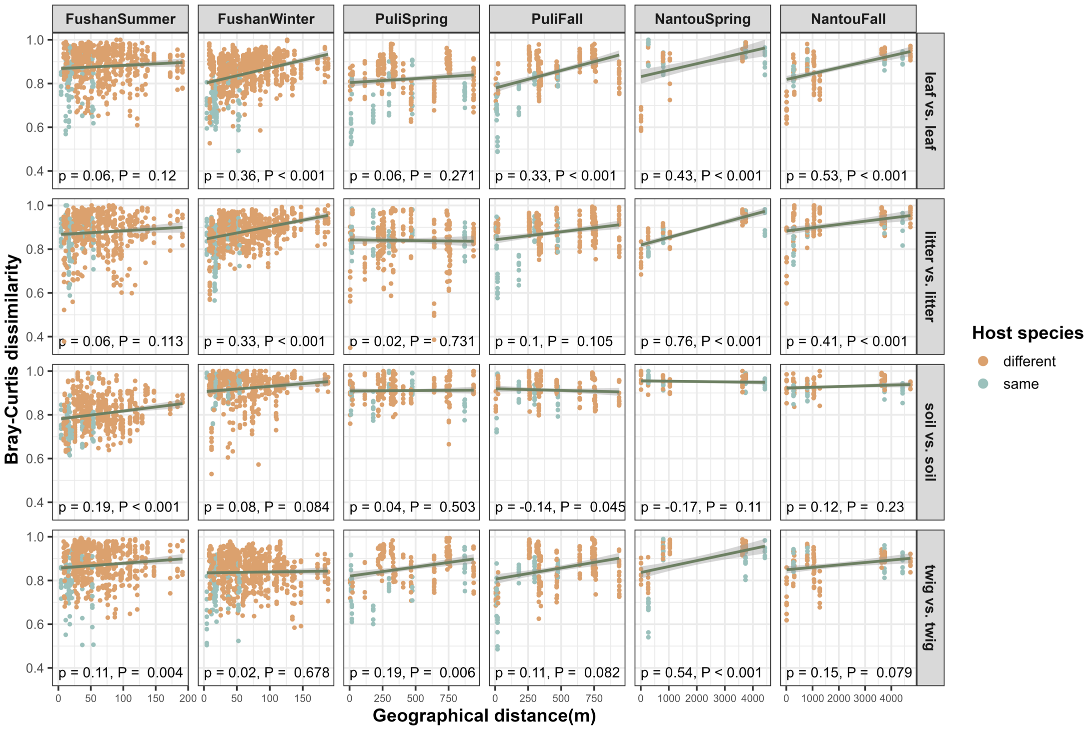

Fig. S8 Bray-Curtis dissimilarity of samples between sites. The boxplots represent the Bray-Curtis dissimilarity index for pairs of samples collected from different sites. Colors indicate whether the comparisons were made within the same habitat (e.g., leaf vs. leaf, litter vs. litter) or between different habitats (e.g., leaf vs. litter, leaf vs. soil).

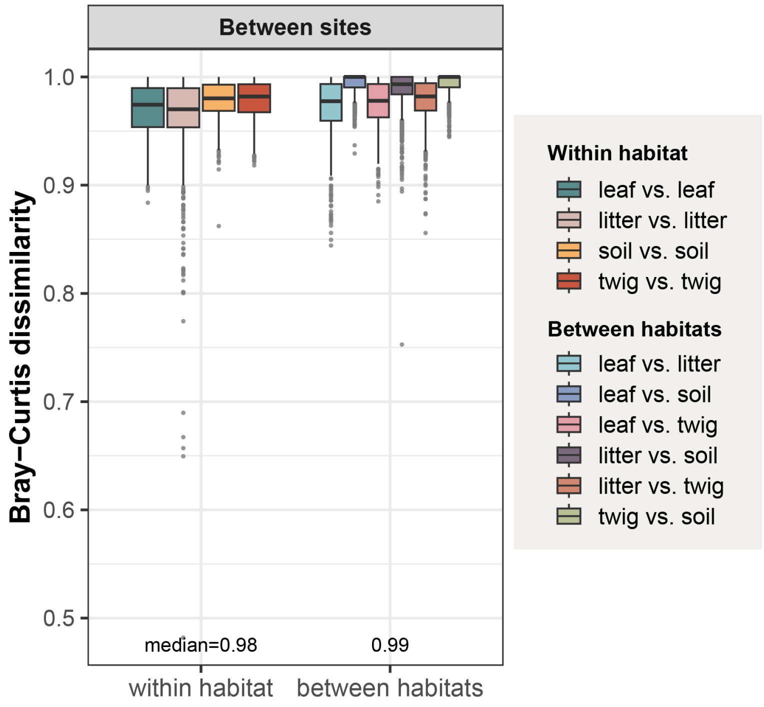

Fig. S9 Variations in mycobiome composition at the genus level across habitats, altitude (a), and precipitation (b). The plots are calculated using Bray-Curtis distance and visualized through Non-metric Multidimensional Scaling (NMDS) and Principal Coordinates Analysis (PCoA), respectively. The PCoA plot explains 14.3% of the variation in the dataset. (a) Shapes indicate host species, and colors represent sample altitudes (meters). (b) Shapes represent different habitats, with colors corresponding to daily (left) and monthly (right) precipitation levels (mm). (c) Shapes denote different habitats, with colors representing their host species.

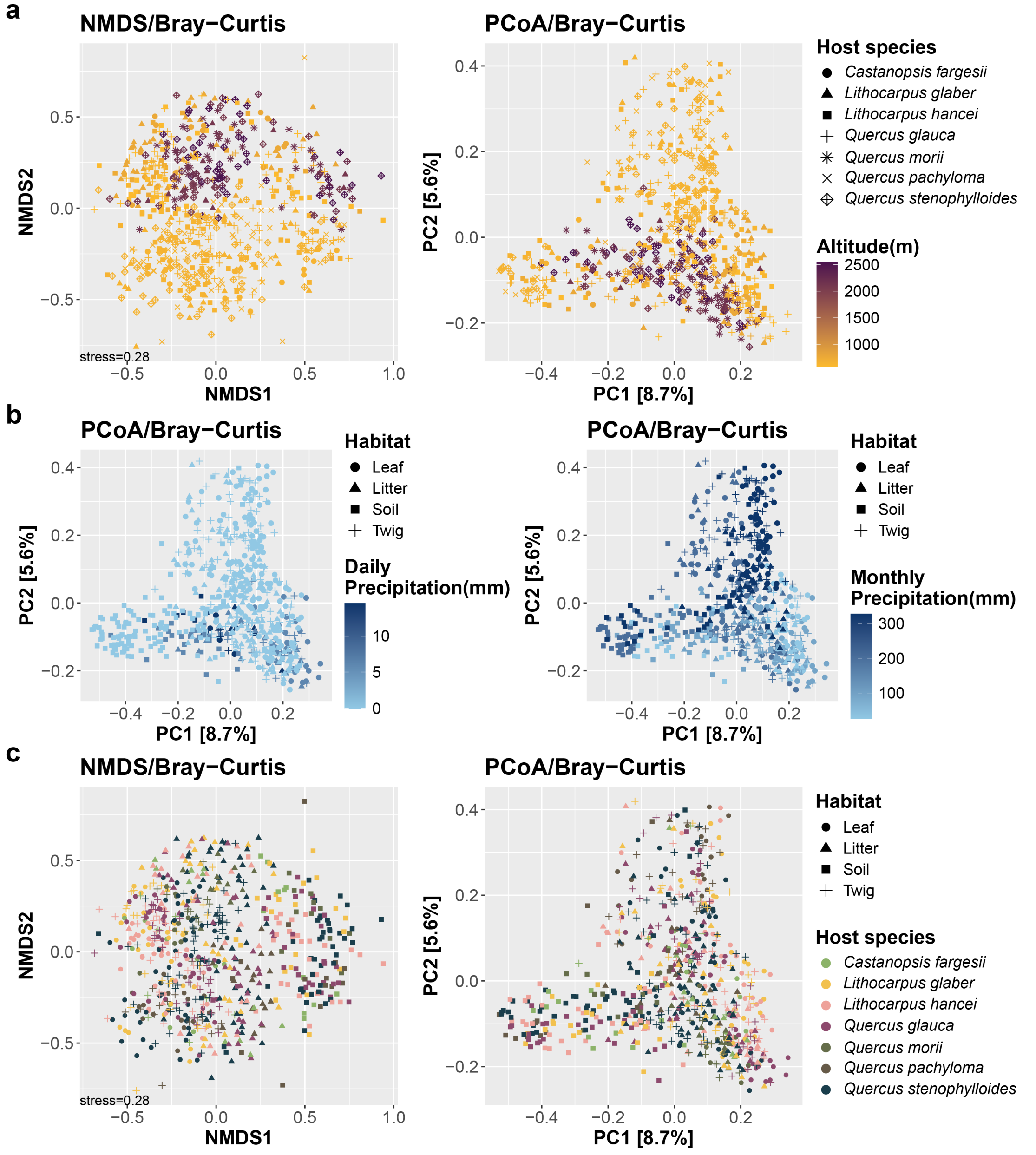

Fig. S10 GDM analysis of non-significant environmental drivers of fungal community across habitats. Fitted I-spine curves from GDM analysis demonstrate the contribution of precipitation and temperature with different temporal scales on the fungal community. The color indicates the habitat. The shaded area represents the error band generated by 1,000 times permutation with 0.7 sampling rate.

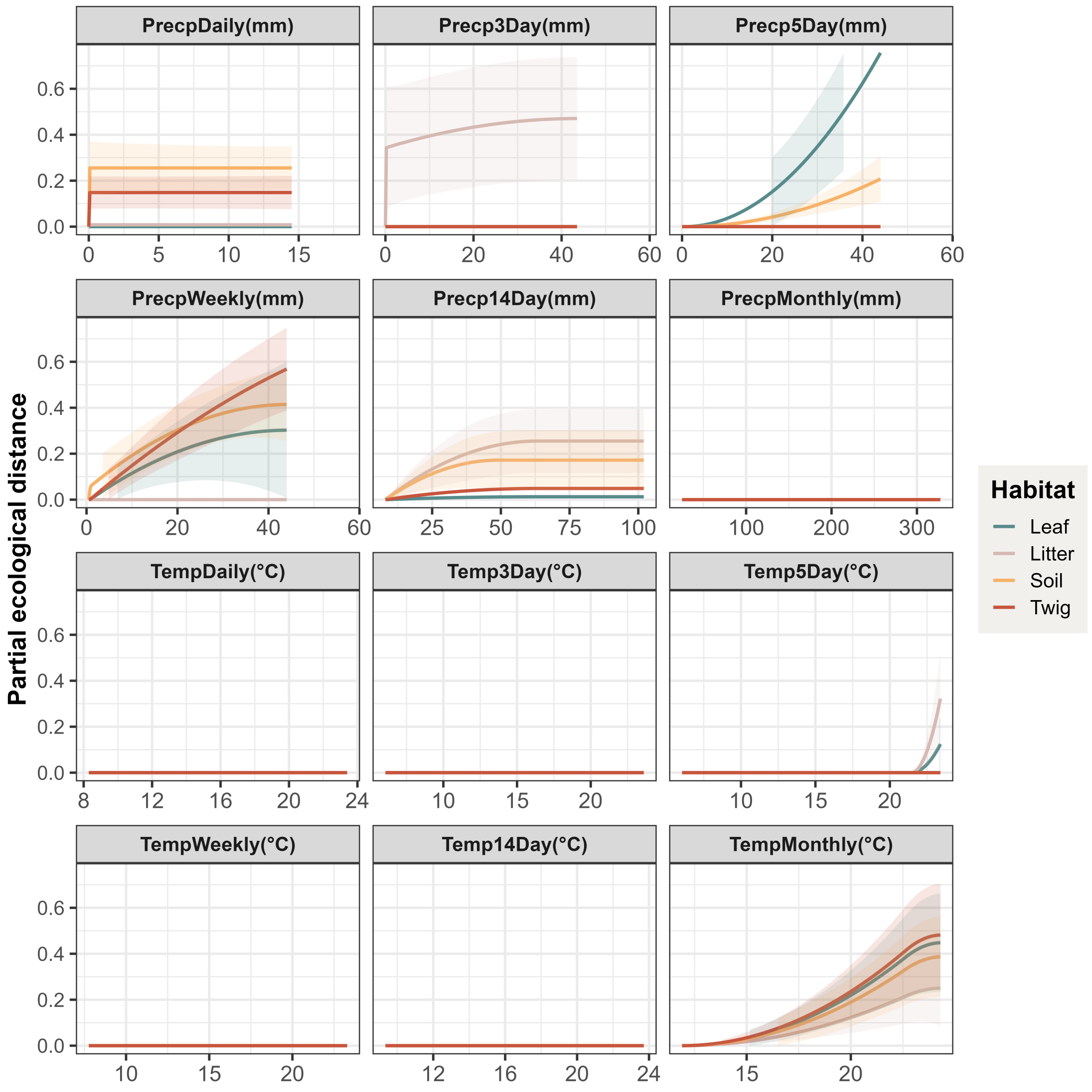

Fig. S11 Relative abundance of ubiquitous ASVs. Each point represents the relative abundance of a specific ASV within a sample. The plot is grouped by habitats (leaf, litter, soil, and twig), with ASVs classified as ubiquitous based on their presence across multiple habitats. The boxplots indicate the distribution of relative abundances for each ASV within each habitat, highlighting the variability and prevalence of these ASVs across different environments.

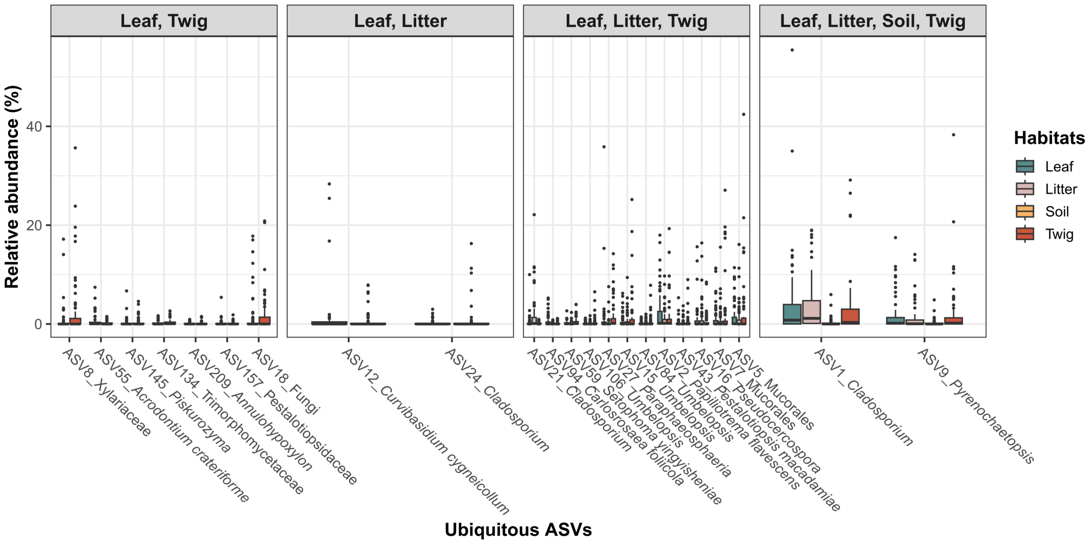

Fig. S12. Random Forest model performance. (a) Line graph illustrating the out-of-bag (OOB) error rate versus the number of trees (ntrees) for both the training set (red) and validation set (blue) showing the model improvement as the number of trees increases. (b) ROC curves show the model's discrimination accuracy across different habitats (soil, leaf, litter, and twig). The area under the curve (AUC) values indicates high predictive accuracy for each habitat.

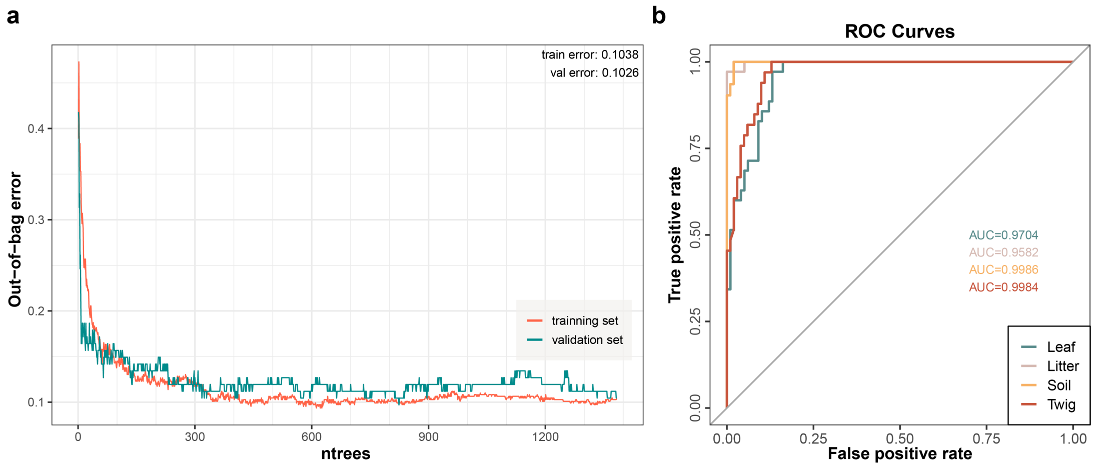

Fig. S13 Co-occurrence networks of ASVs across seasons and habitats. Spearman correlation was utilised to estimate the correlation of mycobiome in different environments. Only strong and significant correlations were graphed (|Spearman's ρ| ≥ 0.6, false positive adjusted p-value < 0.05). The edge colour represents a positive (blue) or negative (red) correlation. The node colour denotes the module it was clustered with.

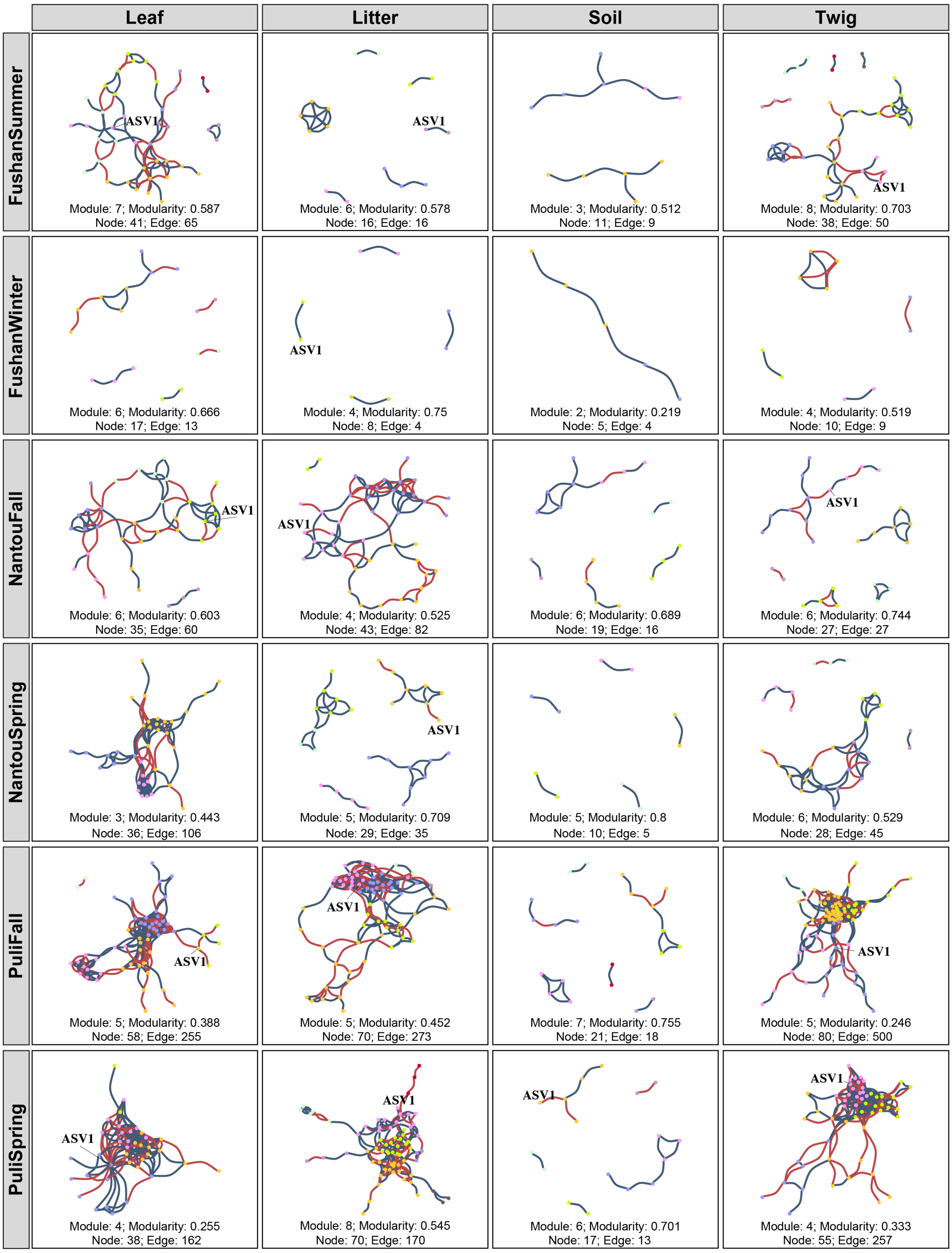

Fig. S14. Correlation between network properties and the normalised stochasticity ratio (NST). Spearman's rank correlation coefficient (ρ) was used to assess the strength of the relationships. Colors represent different seasons, and shapes indicate habitat types.

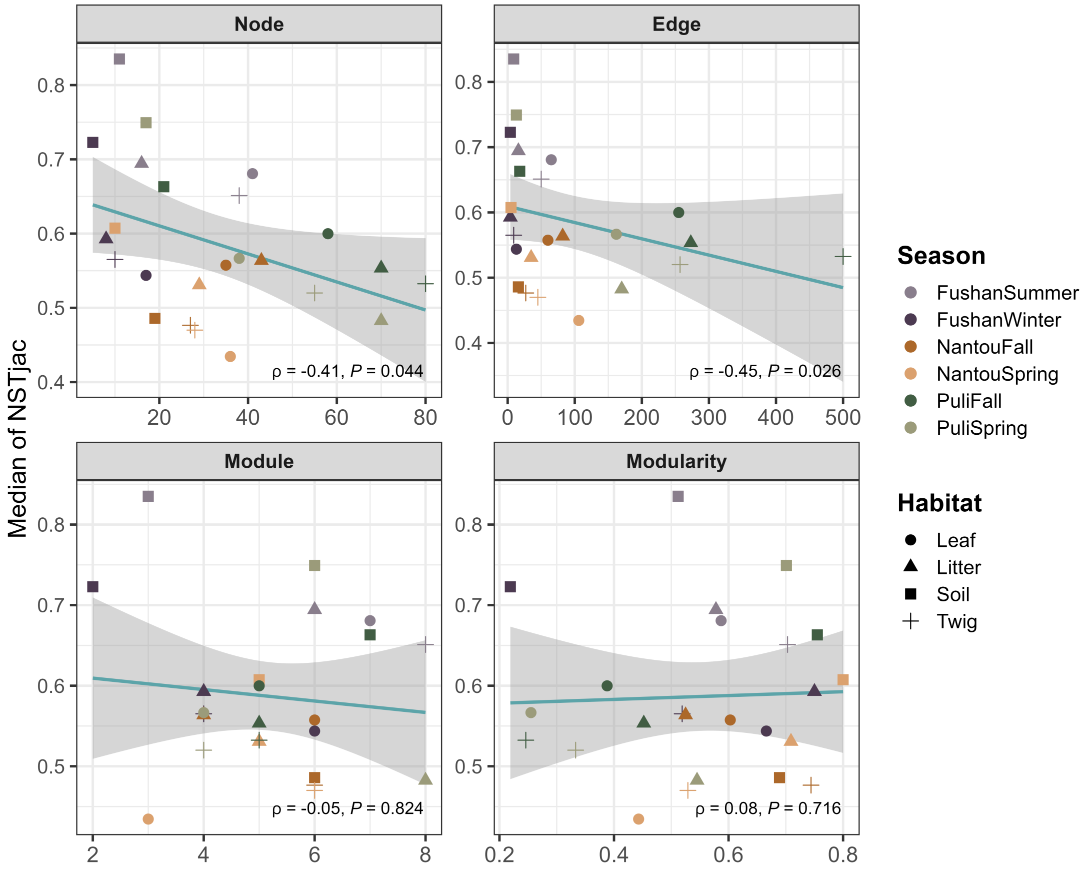

Fig. S15. Proportional changes in trophic modes across co-occurrence networks in 24 different environments. Each point represents the proportion of trophic mode nodes within the networks for leaf, litter, soil, and twig habitats across different seasons and sites.

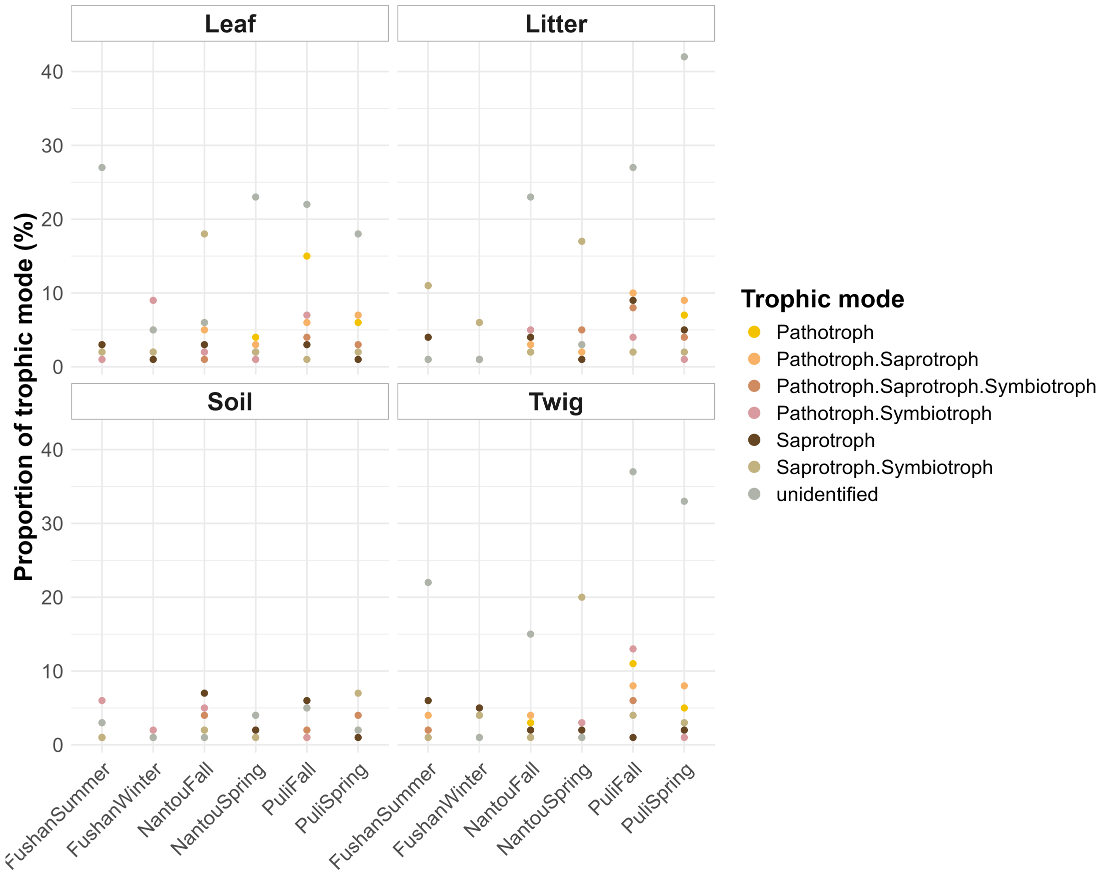

Fig. S16 The connectively within and among modules of vertices in the co-occurrence network. Zi: connectivity within module; Pi: connectivity among modules. The color represents their trophic mode.

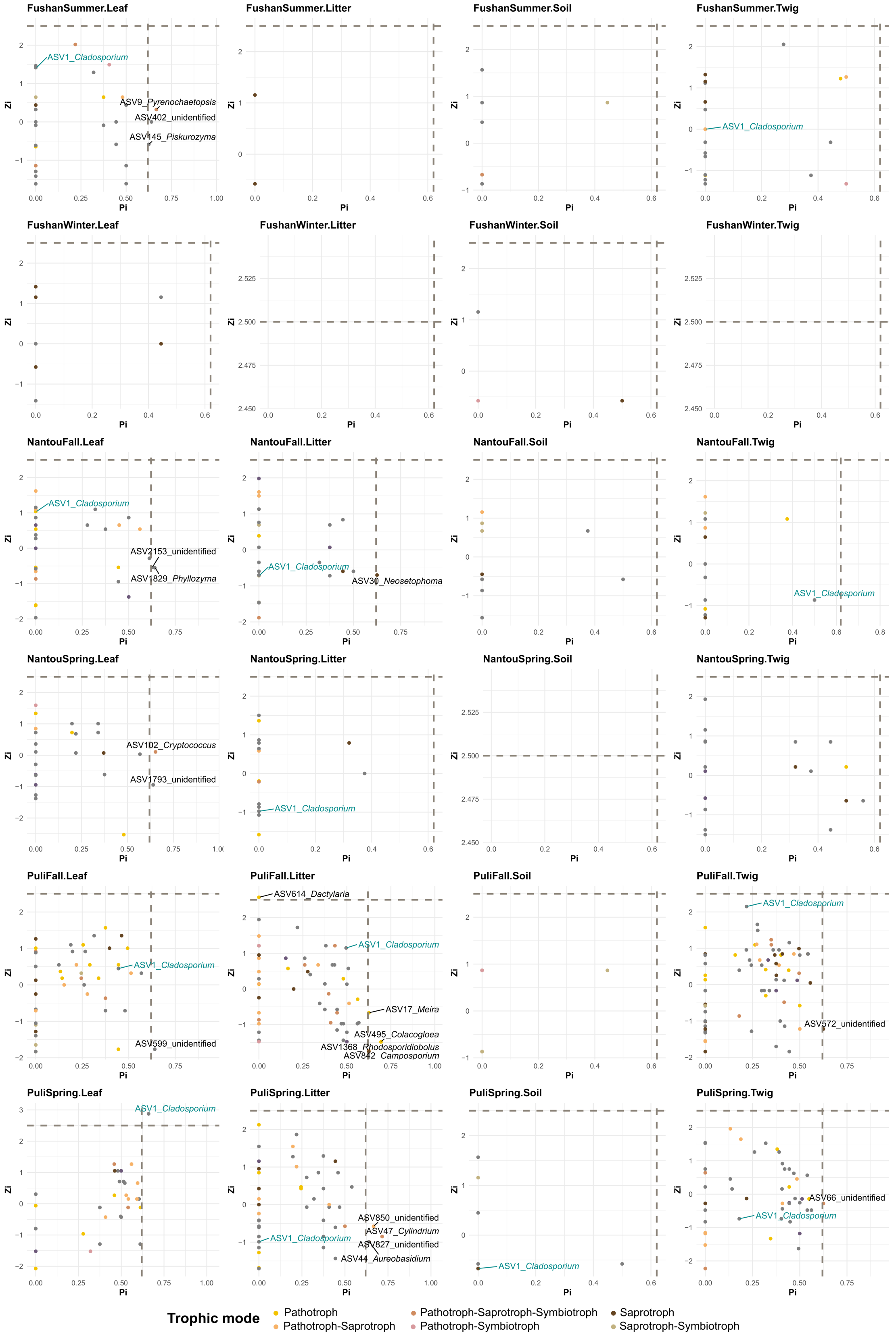

Supplement: Supplementary file 1 — Supplementary Material 1 [file 40793_2025_683_MOESM1_ESM.pdf]
